# An investigation of the flexural behaviour of large-span prestressed and steel-reinforced concrete slabs

**DOI:** 10.1038/s41598-023-37137-6

**Published:** 2023-07-03

**Authors:** Tiancheng Han, Shuting Liang, Xiaojun Zhu, Wenkang Wang, Jian Yang

**Affiliations:** 1 School of Civil Engineering, Southeast University, Nanjing, 210096 China; 2 Key Laboratory of Concrete and Pre-Stressed Concrete Structures, Ministry of Education, Nanjing, 210096 China; 3 Architecture Design and Research Institute Ltd, Southeast University, Nanjing, 210096 China

**Keywords:** Engineering, Civil engineering

## Abstract

The prestressed and steel-reinforced concrete slab (PSRCS) is an innovative composite structural member offering high load capacity and stiffness and exceptional anti-crack performance, making it a leading trend in composite structures. This paper presents the derived calculation formulas for bearing capacity, section stiffness, mid-span deflection of PSRCS. Additionally, a numerical analysis of PSRCS is conducted using ABAQUS software, with several models created to systematically investigate bearing capacity, section stiffness, anti-crack performance, and failure mode. Concurrently, PSRCS member parameters are analyzed for optimal design, and the results of finite element (FE) calculations are compared with theoretical formula calculations. The results demonstrate that PSRCS exhibits superior load capacity, section stiffness, and anti-crack performance comparing to conventional slabs. The parametric analysis offers optimal design for each parameter and presents the corresponding recommended span-to-depth ratios for various spans in PSRCS applications.

## Introduction

Exploiting urban underground resources is a highly effective method for addressing the significant conflict between urban development and limited land availability. Thoughtful planning, development, and use of underground space can substantially support and bolster urban development strategies, emphasizing sustainability worldwide^1,2^. At the same time, depth and span requirements remain crucial factors in underground space design. However, depth and span are mutually exclusive, making it difficult to balance bearing capacity, crack control, and functional usage in large-span underground structures^3,4^. Consequently, traditional practices, such as the underground framework support system at the Messe Bahnhof station in Germany, provide stability and high bearing capacity. Yet, they require numerous columns and have a smaller span, significantly impacting visibility and spatial efficiency^5^. Most subway tunnels and underground stations utilize arch structures, which improve performance over time as they bear only compressive forces internally, compacting the material. Nevertheless, arch structures present considerable limitations in functional usage, such as clearance and short-side span, and pose maintenance challenges^6,7^.

Conventional structural forms face challenges in satisfying both load-bearing capacity and crack control requirements when designing large-span underground structures. To address this issue, this paper introduces a suitable structural form for such structures, specifically, the prestressed and steel-reinforced concrete slab (PSRCS). The PSRCS comprises an H-beam, prestressed tendons, longitudinal reinforcement, and stirrup cages. In this novel structure, the H-beam and longitudinal reinforcement act as the primary load-bearing components, while prestressed tendons control overall crack development and distribution. The H-beam is arranged at intervals within the slab, and stirrup cages are placed between adjacent H-beam elements to ensure uniform sectional stiffness distribution and provide positioning and fixation for the prestressed tendons.

PSRCS represents a novel composite structural form for slabs, which currently lacks research and engineering applications in terms of its flexural performance. Reinforced concrete slabs, prestressed concrete slabs, steel-reinforced concrete slabs, and prestressed steel-reinforced concrete beams are examples of similar flexural members with established research foundations. Since^8^ conducted vertical flexural performance tests on simply supported reinforced concrete slabs with varying spans and examined the failure modes and mechanical behavior of composite beam-slab systems, researchers have been investigating the flexural performance of reinforced concrete slabs from structural to material levels. Abdal et al. and Ibrahim et al.^9,10^ incorporated short carbon fibers into reinforced concrete one-way slabs and analyzed the influence of these additives on the flexural performance of the slabs. Turco et al.^11^ examined the impact of the width and thickness of composite material layers on the flexural performance of reinforced concrete beam slabs. Wagner and Gruttmann^12^ investigated the selection of boundary conditions and the influence of nonlinear material parameter behavior on reinforced concrete ribbed slab systems, addressing the potential for altering material models during the loading process and the associated elasto-plastic behavior, as well as suggesting a proportional adjustment method for the model loading process. Honarvar et al.^13^ utilized experimental data and finite element models to assess the structural performance of prefabricated ultra-high strength reinforced concrete ribbed slab systems and optimized the design methodology for these systems. To enhance the strength and crack resistance of reinforced concrete slab structures, researchers integrated prestressing tendons, shaped steel, and other components into reinforced concrete slabs to create composite slabs and investigated their related vertical load-bearing performance. Zhang et al.^14^ introduced a nonlinear analysis method for determining the limit stress of unbonded reinforcement in prestressed concrete slab systems and presented a novel calculation approach for the flexural performance of these systems using static load tests and theoretical analysis. da Silva et al.^15^ suggested slab, bar, and interface finite element formulations for numerically simulating prestressed concrete beam slabs, emphasizing the inclusion of interface elements that can emulate potential sliding between concrete and prestressed tendons, as well as the stress and strain functions within the reinforcement bundles during vertical loading. Hou et al.^16^ assessed the flexural performance of prefabricated prestressed concrete bridge deck panels, examining the influence of crucial design parameters on the combined beam-slab flexural performance through monotonic loading tests. da Rocha Almeida et al.^17^ investigated an analytical method for steel–concrete composite beams with externally applied prestress and evaluated the structural performance of these prestressed steel–concrete composite beams. Oukaili et al.^18^ introduced a nonlinear analysis method to assess the performance of post-tensioned concrete flexural members with unbonded internal tendons, validating the proposed method through experimental results and finite element models. Research on steel–concrete composite slab systems remains relatively limited. Ayhan et al., Gopinath et al. and Wang et al.^19–21^ examined the flexural and bond performance of cold-formed thin-walled steel–concrete composite slabs, while^22–24^ concentrated on the flexural performance, working performance, and associated influencing factors of steel–concrete hidden beam slabs. Although direct research on PSRCS is currently scarce, a relatively mature research foundation exists for prestressed and steel-reinforced concrete beams (PSRCB), which exhibit similarities with PSRCS. In recent years, researchers have extensively studied the performance and design methods of PSRCB, offering a more comprehensive understanding of this type of structure. Du et al.^25^ explored the flexural performance of PSRCB and the bond-slip effects between steel and concrete, conducted static load tests on PSRCB, revised the existing calculation formula for PSRCB's positive cross-section bearing capacity, and obtained bond-slip influence coefficients for different component parts through regression analysis using existing experimental data. Yao and Xiong^26^ examined the limit values of flexural deformation performance indices for PSRCB, summarized the complete failure characteristics of PSRCB in bending, and provided corresponding macroscopic damage descriptions. Li and Yu et al.^27,28^ combined prestressing technology with prefabrication techniques in steel-reinforced concrete structures, proposed partially prefabricated prestressed steel-reinforced concrete (PPPSRC) beams, conducted flexural performance tests, and analyzed the effects of relevant parameters on the failure modes and cross-sectional stress development of PPPSRC beams. Chen et al., Fu et al. and Yao and Xiong^29–31^ performed experimental studies on the vertical load-bearing performance and failure mechanisms of prestressed steel-reinforced concrete frames. Research results indicate that PSRCB structures can effectively control crack width, enhance the normal usage performance of the structure, fully utilize the strengths of steel and concrete, and maximize the benefits of different materials.

The aforementioned research on the vertical load-bearing performance of similar flexural components indicates that whether it involves reinforced concrete ribbed slabs, waffle slabs, prestressed concrete composite slabs, or cold-formed thin-walled steel–concrete composite slabs, none can achieve both high load-bearing capacity and effective crack control simultaneously. The integration of prestress and H-beams is often employed only in frame beam-column systems, with no systematic theoretical research or engineering applications for PSRCS in directly load-bearing components such as slabs. Consequently, investigating the vertical load-bearing performance of PSRCS can broaden the research scope of prestressed steel-reinforced concrete composite structures. Simultaneously, its advantages of high load-bearing capacity and superior crack resistance make it suitable for the design and application of large-span underground structures, providing a theoretical foundation and design reference for future engineering projects.

This study examines the load-bearing mechanism of PSRCS under vertical loads. From a theoretical analysis standpoint, the paper presents calculation methods for the bearing capacity, stiffness, and deflection of PSRCS cross-sections. Utilizing ABAQUS finite element software, a comparative analysis highlights the advantages of PSRCS over traditional slab construction systems. A parametric analysis is also conducted to investigate the bearing capacity of PSRCS under various conditions and the stress changes within internal components. Furthermore, the study offers optimal designs for diverse spans and slab thicknesses.

## Theoretical calculations of bearing capacity, section stiffness, and deflection of PSRCS

### Calculation method of bearing capacity of PSRCS

**Figure 1:**
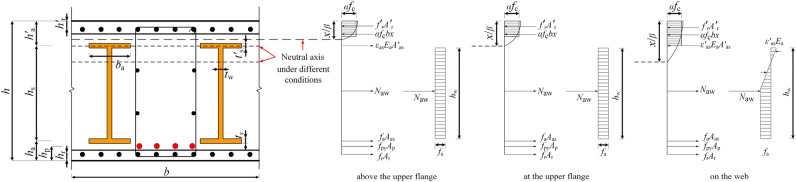
The neutral axis of PSRCS under different conditions.

The unique mechanism of PSRCS differentiates it from traditional unidirectional slabs. For calculations, a cross-section is considered, which includes an H-beam, prestressed tendons, and longitudinal reinforcement under a unit width along the short side of the PSRCS. The following calculation relies on the plane section hypothesis and^32^’s research. The complexity of the PSRCS stems from factors like the tensile reinforcement ratio, steel content of the H-beam, H-beam eccentricity, and the height of the prestressed tendons. Consequently, the entire co-working process exhibits complexity. Depending on the neutral axis positions, the PSRCS can be classified into three cases, as depicted in Fig. 1.

Where *x/β* represents the height of the compression zone;* h* is the section height of PSRCS; *b* is the calculation width of the cross section; *b*_a_ is the width of flange of H-beam; *h*_s_ is the height between the centers of the upper and lower flanges of the H-beam; *h*_w_ is the height of H-beam web; *h*_a_ and *h*^*'*^_a_ are the distances from the centers of the lower and upper flanges of the H-beam to the tension and compression edges of the cross-section, respectively; *h*_p_ is the distance from prestressed tendons to the tension edge of the cross-section; *h*_r_ is the distance between the tension reinforcement and the lower edge of the cross-section; *t*_s_ and *t*^*'*^_s_ are the thicknesses of the lower and upper flanges of the H-beam, respectively; *t*_w_ is the web thickness of H-beam; *f*_r_, *f*_py_, and *f*_a_ are the design yield strength values of the tensile reinforcement, prestressed tendons, and H-beam, respectively; *f*_c_ is the design compressive strength value of the concrete; *A*_r_ is the area of ordinary reinforcement of concrete in the tensile zone; *A*^*'*^_r_ is the area of ordinary reinforcement of concrete in the compressive zone; *A*_p_ is the area of prestressed tendons; *A*_as_ and *A*^*'*^_as_ are the area of bottom flange and upper flange of H-beam; *A*_aw_ is the area of H-beam web; *N*_aw_ is the axial force of H-beam web; *ε*_as_ is the tensile strain at the center of the upper flange of H-beam; *E*_a_ is the elastic modulus of the H-beam.

1. In PSRCS, when the H-beam is asymmetrically arranged (with a downward offset) or when the steel ratio is relatively low, the neutral axis may lies above the upper flange of the H-beam. The upper flange, web, bottom flange of the H-beam, as well as tensile reinforcement and prestressed tendons, are assumed to be in tension, and the compressed concrete in the compression zone is partially crushed and ceases to function. For simplicity in calculation, it is considered that the compressed concrete in the compression zone has reached its yield strength. At this point, the analysis focuses on the steel flange near the neutral axis, while the stress states of the other steel components, such as longitudinal reinforcement and prestressed tendons, are considered relatively simple and assumed to have reached their yield strength. Based on the stress balance in the section, when *ε*_as_ is smaller than *ε*_a_ and *x/β* is smaller than *h*^*'*^_a_, the relative height of the concrete compressive zone can be calculated as follows:
  1 $$\left\{ \begin{gathered} \alpha f_{{\text{c}}} bx + f_{{\text{r}}}^{\prime } A_{{\text{r}}}^{\prime } = f_{{\text{r}}} A_{{\text{r}}} + f_{{{\text{py}}}} A_{{\text{p}}} + f_{{\text{a}}} A_{{{\text{as}}}} + \varepsilon_{{{\text{as}}}} E_{{\text{a}}} A_{{{\text{as}}}}^{\prime } + N_{{{\text{aw}}}} \hfill \\ N_{{{\text{aw}}}} = f_{{\text{a}}} A_{{{\text{aw}}}} \hfill \\ M_{{{\text{aw}}}} = f_{{\text{a}}} A_{{{\text{aw}}}} \left( {h_{{\text{a}}}^{\prime } + t_{{\text{s}}}^{\prime } + h_{{\text{w}}} /2 - x/\beta } \right) \hfill \\ \varepsilon_{{{\text{as}}}} = \frac{{\varepsilon_{{{\text{cu}}}} \left( {h_{{\text{a}}}^{\prime } + t_{{\text{s}}}^{\prime } /2 - x/\beta } \right)}}{x/\beta } \hfill \\ \end{gathered} \right.$$
  where *x* is the relative height of the compressive zone; *ε*_cu_ represents the ultimate compressive strain of concrete; *M*_aw_ denotes the bending moment of the H-beam web.
  The *α* and *β* are coefficients of the equivalent rectangular stress pattern, and they are obtained by the method in reference^33^.
  2 $$\left\{ \begin{gathered} \alpha = \frac{j}{\beta } = \frac{j}{2(1 - k)} \hfill \\ \beta = 2\left( {1 - k} \right) \hfill \\ \end{gathered} \right.$$
  The coefficient of* j* and *k* are determined by the following formula (3):
  3 $$j = \frac{{\int_{0}^{{\varepsilon_{{{\text{cu}}}} }} {\sigma_{{\text{c}}} \left( \varepsilon \right) \cdot d\varepsilon } }}{{f_{{\text{c}}} \varepsilon_{{{\text{cu}}}} }}\quad k{ = }\frac{{\int_{0}^{{\varepsilon_{{{\text{cu}}}} }} {\sigma_{{\text{c}}} \left( \varepsilon \right) \cdot \varepsilon \cdot d\varepsilon } }}{{j \cdot f_{{\text{c}}} \varepsilon^{2}_{{{\text{cu}}}} }}$$
  The formula of bearing capacity of PSRCS is calculated as follows:
  4 $$\begin{gathered} M \le \alpha f_{{\text{c}}} bx\left( {x/\beta - x/2} \right) + f_{{\text{r}}}^{{\prime }} A_{{\text{r}}}^{{\prime }} \left( {x/\beta - h_{{\text{r}}}^{{\prime }} } \right) + \varepsilon_{{{\text{as}}}} E_{{\text{a}}} A_{{{\text{as}}}}^{{\prime }} \left( {h_{{\text{a}}}^{{\prime }} + t_{{\text{s}}}^{{\prime }} /2 - x/\beta } \right) + M_{{{\text{aw}}}} \hfill \\ + f_{{\text{a}}} A_{{{\text{as}}}} \left( {h - h_{{\text{a}}} - t_{{\text{s}}} /2 - x/\beta } \right) + f_{{{\text{py}}}} A_{{\text{p}}} \left( {h - h_{{\text{p}}} - x/\beta } \right) + f_{{\text{r}}} A_{{\text{r}}} \left( {h - h_{{\text{r}}} - x/\beta } \right) \hfill \\ \end{gathered}$$
2. When the neutral axis is situated on the upper flange of the H-beam, it is assumed that the stress on the upper flange of the section steel is zero. Based on the stress balance of the cross-section, Eqs. (1) and (2) are simplified, and the calculation formula for the relative height of the compressed concrete area x is as follows:
  5 $$\left\{ \begin{gathered} \alpha f_{{\text{c}}} bx + f_{{\text{r}}}^{{\prime }} A_{{\text{r}}}^{{\prime }} = f_{{\text{r}}} A_{{\text{r}}} + f_{{{\text{py}}}} A_{{\text{p}}} + f_{{\text{a}}} A_{{{\text{as}}}} + N_{{{\text{aw}}}} \hfill \\ N_{{{\text{aw}}}} = f_{{\text{a}}} A_{{{\text{aw}}}} \hfill \\ M_{{{\text{aw}}}} = f_{{\text{a}}} A_{{{\text{aw}}}} \left( {h_{{\text{a}}}^{{\prime }} + t_{{\text{s}}}^{{\prime }} + h_{{\text{w}}} /2 - x/\beta } \right) \hfill \\ \end{gathered} \right.$$
  The formula of bearing capacity of PSRCS is calculated as follows:
  6 $$\begin{gathered} M \le \alpha f_{{\text{c}}} bx\left( {x/\beta - x/2} \right) + f_{{\text{r}}}^{{\prime }} A_{{\text{r}}}^{{\prime }} \left( {x/\beta - h_{{\text{r}}}^{{\prime }} } \right) + f_{{\text{a}}} A_{{{\text{as}}}} \left( {h - h_{{\text{a}}} - t_{{\text{s}}} /2 - x/\beta } \right) \hfill \\ + f_{{{\text{py}}}} A_{{\text{p}}} \left( {h - h_{{\text{p}}} - x/\beta } \right) + f_{{\text{r}}} A_{{\text{r}}} \left( {h - h_{{\text{r}}} - x/\beta } \right) + M_{{{\text{aw}}}} \hfill \\ \end{gathered}$$
3. In certain cases, when the neutral axis is located within the web of the H-beam, it is assumed that the upper flange of the H-beam experiences compression, the H-beam web undergoes a combination of compression and tension, and the lower flange of the H-beam, the longitudinal reinforcement, and the prestressed tendons are subjected to tension. In accordance with the stress balance, when *h*^*'*^_a_ + *t*^*'*^_s_ < *x*/*β* < *h*^*'*^_a_ + *h*_w_ + *t*^*'*^_s_, the relative height of the compressive zone in the concrete is calculated as follows:
  7 $$\left\{ \begin{gathered} \alpha f_{{\text{c}}} bx + f_{{\text{r}}}^{{\prime }} A_{{\text{r}}}^{{\prime }} { + }\varepsilon_{{{\text{as}}}}^{{\prime }} E_{{\text{a}}} A_{{{\text{as}}}}^{{\prime }} { = }f_{{\text{r}}} A_{{\text{r}}} + f_{{{\text{py}}}} A_{{\text{p}}} + f_{{\text{a}}} A_{{{\text{as}}}} + N_{{{\text{aw}}}} \hfill \\ N_{{{\text{aw}}}} = f_{{\text{a}}} \left[ {2x/\beta - \left( {h_{{\text{w}}} + 2h_{{\text{a}}}^{{\prime }} + 2t_{{\text{s}}}^{{\prime }} } \right)} \right]t_{{\text{w}}} \hfill \\ M_{{{\text{aw}}}} = \frac{1}{2}f_{{\text{a}}} \left[ {x/\beta - h_{{\text{a}}}^{{\prime }} - t_{{\text{s}}}^{{\prime }} } \right]^{2} t_{{\text{w}}} + \frac{1}{2}f_{{\text{a}}} \left[ {h_{{\text{w}}} - x/\beta + h_{{\text{a}}}^{{\prime }} + t_{{\text{s}}}^{{\prime }} } \right]^{2} t_{{\text{w}}} \hfill \\ \end{gathered} \right.$$
  where *ε*^*'*^_as_ is the compressive strain of the upper flange of H-beam; *ε*^*'*^_a_ is the yield compressive strain of H-beam.
  The formula of bearing capacity of PSRCS is calculated as follows:
  8 $$\begin{gathered} M \le \alpha f_{{\text{c}}} bx\left( {x/\beta - x/2} \right) + f_{{\text{r}}}^{{\prime }} A_{{\text{r}}}^{{\prime }} \left( {x/\beta - h_{{\text{r}}}^{{\prime }} } \right) + \varepsilon_{{{\text{as}}}} E_{{\text{a}}} A_{{{\text{as}}}}^{{\prime }} \left( {x/\beta - h_{{\text{a}}}^{{\prime }} - t_{{\text{s}}}^{{\prime }} /2} \right) + M_{{{\text{aw}}}} \hfill \\ + f_{{\text{r}}} A_{{\text{r}}} \left( {h - h_{{\text{r}}} - x/\beta } \right) + f_{{{\text{py}}}} A_{{\text{p}}} \left( {h - h_{{\text{p}}} - x/\beta } \right) + f_{{\text{a}}} A_{{{\text{as}}}} \left( {h - h_{{\text{a}}} - t_{{\text{s}}} /2 - x/\beta } \right) \hfill \\ \end{gathered}$$

### Calculation method of section stiffness and deflection of PSRCS

**Figure 2:**
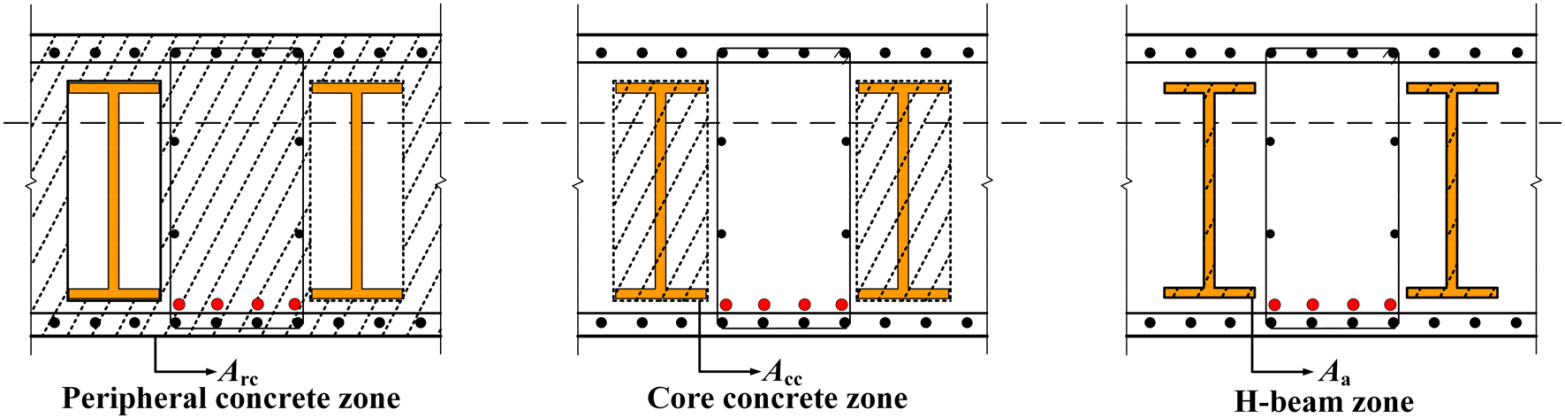
Effective working sections of PSRCS.

Referring to Liu's study on the stiffness of steel reinforced concrete structures^34^, this paper presents a method for calculating the stiffness of PSRCS under various working stages, as illustrated in Fig. 2.

1. When the upper load is less than the cracking load, the PSRCS is in the full-section working state, and its section stiffness *B*_e_ is considered as the summation of the concrete stiffness *B*_c_, the longitudinal reinforcement stiffness *B*_r_, the prestressed tendons stiffness *B*_py_ and the H-beam stiffness *B*_a_. And the overall stiffness *B*_E_ is as follows:
  9 $$\begin{gathered} B_{{\text{e}}} = 0.85\left( {B_{{\text{c}}} + B_{{\text{r}}} + B_{{{\text{py}}}} } \right) + B_{{\text{a}}} { = }0.85E_{{\text{c}}} I_{{\text{c}}} + 0.85E_{{\text{r}}} I_{{\text{r}}} + 0.85E_{{{\text{py}}}} I_{{{\text{py}}}} \\ + E_{{\text{a}}} \left[ {I_{{\text{a}}} + A_{{\text{a}}} \left( {h_{{\text{s}}} /2 + h_{{\text{a}}}^{{\prime }} - x/\beta } \right)^{2} } \right] \\ \end{gathered}$$
  where *E*_c_ is the concrete modulus, *I*_c_ is the moment of inertia of the concrete section, *I*_a_, *I*_r_, *I*_py_ are the moment of inertia of the H-beam, longitudinal reinforcement, and prestressed tendons respectively, *A*_a_ is the area of the H-beam.
2. When the upper load exceeds the cracking load, the peripheral concrete at the bottom experiences cracking and progressively ceases to contribute to the structural performance. Consequently, the neutral axis shifts upward, and the section stiffness comprises the combined stiffness of the core-restrained concrete *B*_cc_, peripheral concrete stiffness *B*_rc_ and H-beam stiffness *B*_a_. The calculation of *B*_rc_ is based on the relevant equations for computing ordinary reinforced concrete structures in the "Concrete Structure Design Code" (GB50010-2010)^35^. Taking into account the overall force balance, the section steel and prestressed tendons are considered as equivalent longitudinal reinforcements, leading to an update and replacement of the equation. The overall stiffness *B*_u_ can be expressed as follows:
  10 $$\left\{ \begin{gathered} B_{{\text{u}}} = B_{{{\text{rc}}}} + B_{{{\text{cc}}}} + B_{{\text{a}}} \hfill \\ B_{{{\text{rc}}}} = \frac{{0.85E_{{\text{c}}} I_{{{\text{rc}}}} }}{{\frac{{M_{{{\text{cr}}}} }}{{M_{{\text{k}}} }} + \left( {1 - \frac{{M_{{{\text{cr}}}} }}{{M_{{\text{k}}} }}} \right)\left[ {1.0 + \frac{0.21}{{\frac{{E_{{{\text{av}}}} }}{{E_{{\text{c}}} }}\left( {\rho_{{\text{r}}} + \rho_{{{\text{py}}}} } \right)}} - 0.7} \right]}} \hfill \\ B_{{{\text{cc}}}} = E_{{\text{c}}} \left[ {I_{{{\text{cc}}}} + A_{{{\text{cc}}}} \left( {y_{{{\text{cc}}}} - x/\beta } \right)^{2} } \right] \hfill \\ B_{{\text{a}}} = E_{{\text{a}}} \left[ {I_{{\text{a}}} + A_{{\text{a}}} \left( {h_{{\text{s}}} /2 + h_{{\text{a}}}^{{\prime }} - x/\beta } \right)^{2} } \right] \hfill \\ \end{gathered} \right.$$
  where the coefficient of 0.85 and 0.21 is derived from "Concrete Structure Design Code" (GB50010-2010)^35^ and is used to account for the difference between the design value and characteristic value of concrete strength, ensuring conservative design and high safety levels; *I*_rc_ and *I*_cc_ are the moments of inertia of the peripheral region and the core region; *A*_a_ is the sectional area of H-beam; *y*_cc_ is the distance from the centroid of the concrete core region to the compressive area surface; *M*_cr_ is the cracking moment, which represents the moment when the concrete section of the beam starts to crack, and can be calculated based on the tensile strength of the concrete, the area of reinforcement, and the effective depth of the beam; *M*_k_ is the ultimate moment, indicating the moment when the beam fails due to the yielding of the reinforcement, and is determined by considering the maximum distributed load on the beam, the span of the beam, and the effective depth of the beam; *E*_av_ is the average elastic modulus of H-beam, longitudinal reinforcement and prestressed tendons; *ρ*_r_ and *ρ*_py_ are the reinforcement ratio of longitudinal reinforcement and prestressed tendons, respectively.
  The deflection of PSRCS consists of two following parts: the first part is the deflection *y*_e_ caused under the full working stage, and the second part is the deflection *y*_u_ caused after the concrete cracking. Equation (11) is derived based on the relevant provisions of "Concrete Structure Design Code" (GB50010-2010)^35^ and the references^36,37^, as shown below:
  11 $$y = y_{{\text{e}}} + y_{{\text{u}}} = \lambda \left[ {\frac{{M_{{{\text{cr}}}} l^{2} }}{{B_{1} }} + \mu \frac{{\left( {M_{{\text{k}}} - M_{{{\text{cr}}}} } \right)l^{2} }}{{B_{2} }}} \right]$$
  where *λ* is the deflection coefficient, which is related to the support conditions and the form of upper load, *μ* is the proposed stiffness reduction factor considering the development of concrete cracks and the reduction of the restraint effect of the gradual yielding of the H-beam in the core area of concrete, *l* is the design span of PSRCS.

## Overview of the studied models and the establishment of FE models

### Overview of the studied models

**Figure 3:**
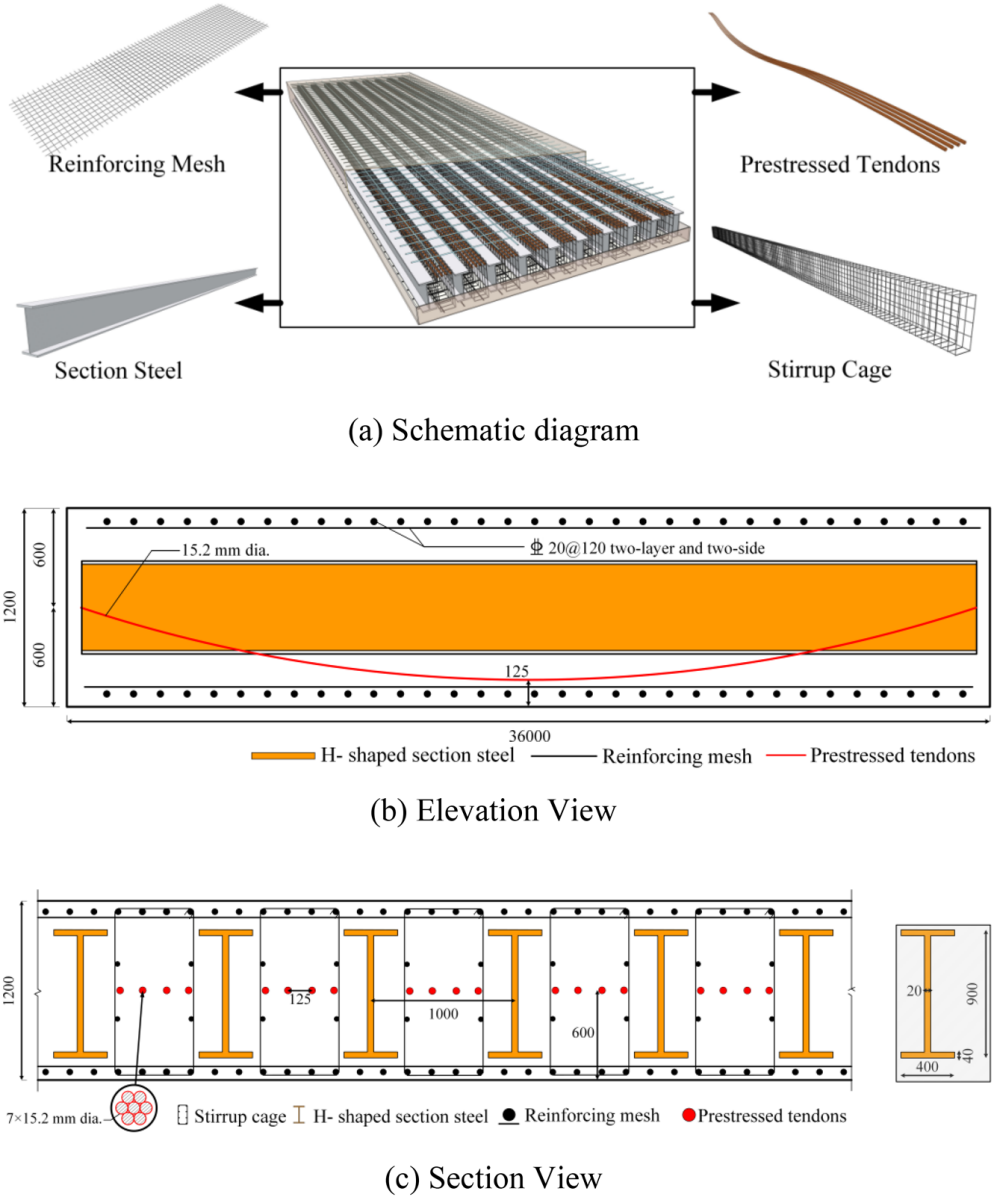
Schematic diagram and details of PSRCS.

As depicted in Fig. 3, the prestressed steel-reinforced concrete slab (PSRCS) is based on an ongoing project located in Nanjing, China. The composition of the internal components of PSRCS has already been discussed in the introduction. The detailed dimensions and specifics of PSRCS can be found in Fig. 3b, c. The design dimensions of PSRCS are 36 m × 12 m × 1.2 m. To investigate its mechanical properties, the support conditions are designed as simply supported, with ten sets of H-beams inside, each measuring 900 mm × 400 mm × 20 mm × 40 mm. There are a total of 36 sets of prestressed tendons designed with a curvilinear linear layout. The lowest point in the mid-span of PSRCS is 125 mm from the bottom of the slab. The supports are positioned at the center of the slab's height to prevent the occurrence of negative bending moments. The longitudinal reinforcements at the top and bottom of the slab are designed with HRB400-grade steel bars with a diameter of 20 mm, spaced 120 mm apart.

11 unique PSRCS samples and 2 comparative samples, including reinforced concrete slab (RCS) and steel-reinforced concrete slab (SRCS), have been modeled, as displayed in Table 1. The finite element (FE) method is employed to investigate the effects of various PSRCS design parameters, such as H-beam eccentricity, span-to-depth ratio, ratios of longitudinal reinforcement and prestressed tendons, and steel content. Two separate values are considered for parameter selection within the model.

**Table 1:** Design cases of FE models.

Model	Eccentricity of steel	Ratio of *LR* (%)	Ratio of *PT* (%)	Steel content (%)	Ratio of span-depth
RCS	–	–	–	–	30
SRCS	–	–	–	1.3	30
PSRCS-1	0	0.2	0.27	1.3	30
PSRCS-2	− 30	0.2	0.27	1.3	30
PSRCS-3	+ 30	0.2	0.27	1.3	30
PSRCS-4	0	0.2	0.27	1.3	27.7
PSRCS-5	0	0.2	0.27	1.3	24
PSRCS-6	0	0.4	0.27	1.3	30
PSRCS-7	0	0.6	0.27	1.3	30
PSRCS-8	0	0.2	0.14	1.3	30
PSRCS-9	0	0.2	0.4	1.3	30
PSRCS-10	0	0.2	0.27	0.93	30
PSRCS-11	0	0.2	0.27	1.81	30

‘–’ means not measured items; *LR* means longitudinal reinforcement; *PT* means prestressed tendons.

‘Eccentricity of steel’ means ‘−30’ means the position of section steel of PSRCS-2 is 30 mm lower than the neutral axis.

### FE model validation

Due to the lack of direct research on PSRCS, it is essential to evaluate the accuracy and reliability of this innovative structure in ABAQUS. In this paper, Zheng's experiments^38^ were utilized to validate the results of the FE model of prestressed composite concrete beams with encased H-steel.

**Figure 4:**
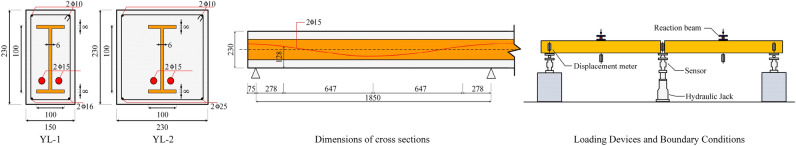
schematic diagram of component cross-sectional dimensions in^38^.

Zheng et al.^38^ designed three sets of prestressed composite concrete beams with encased H-steel specimens, as shown in Fig. 4. The designed concrete strength grade is C40; prestressed tendons utilize steel strands with a tensile strength standard value of *f*_ptk_ = 1860 N/mm^2^, and the controlled tensile stress is *σ*_con_ = 0.75*f*_ptk_, which using bonded prestress technology. H-shaped steel is of Q235 strength grade, symmetrically arranged. The concrete's cubic compressive strength *f*_cu_ = 50.12 N/mm^2^, axial tensile strength *f*_t_ = 3.38 N/mm^2^, and elastic modulus *E*_c_ = 3.25 × 10^4^ N/mm^2^. The measured yield strength *f*_y_, ultimate strength *f*_u_, and yield strain *ε*_y_ of the reinforcement and steel sections are presented in Table 2 of Ref.^35^. The loading method involves using hydraulic jacks to symmetrically apply single-point loads at the mid-span of the two spans of the specimen, with simply supported boundary conditions and adjustable hinge supports at the central support.

To verify the effectiveness and accuracy of the FE modeling method for PSRC-type components compared to actual tests, the details of the establishment of the finite element model in^38^ are presented below. The geometry and dimensions of the finite element model are consistent with the YL-2 component in^38^. Through the mesh sensitivity study, to facilitate computation, the grid size in the span direction is set at 100 mm intervals, while the grid size in the width direction is set at 30 mm intervals. In terms of material properties, the elastic modulus, Poisson's ratios, and yield strengths of concrete and steel are consistent with those in^38^. For the element type, since the component is a steel–concrete composite structure, both concrete and section steel are modeled using C3D8R solid elements to improve accuracy, while rebars and prestressed tendons are modeled using T3D2 truss line elements. In the selection of boundary conditions, to be consistent with the simple support conditions of the experimental component, the boundary conditions at both ends of the model are defined: one end is a fixed support, restricting its translational and out-of-plane rotational movements, while the other end is set as a sliding support, constraining its out-of-plane translation, vertical displacement, and out-of-plane rotation. Displacement loading is selected, and equivalent displacement corresponding to the experimental limit load is applied. Prestressing is treated using the predefined Fields tool^39^. The internal contact interaction of the component is handled using embedded constraints. The calculation process employs static force computation.

**Figure 5:**
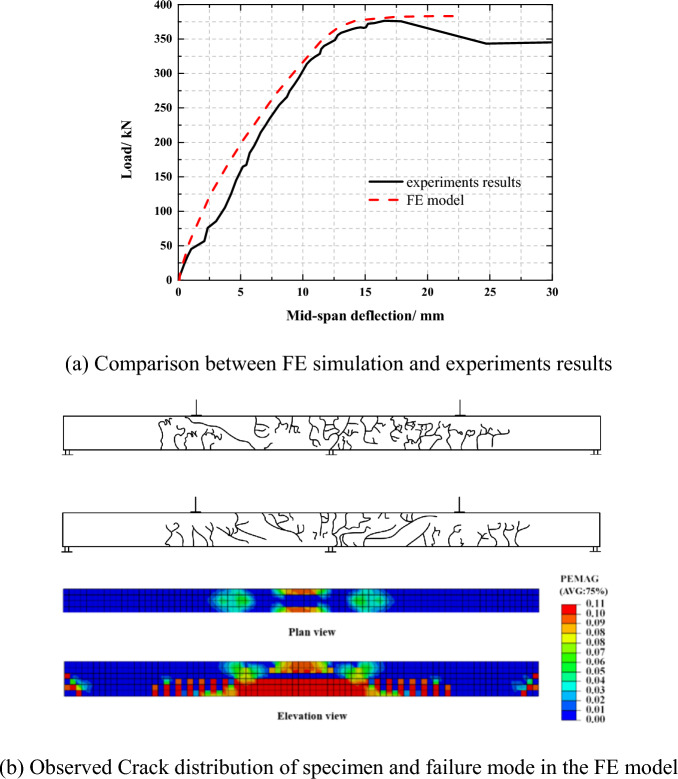
Comparison between the reference test^38^ and FE simulation.

The comparative analysis between ABAQUS results and Zheng's experiments results are shown in Fig. 5. The ABAQUS results and Zheng's experiments results are in good agreement on the overall trend, The ABAQUS model can effectively simulate the mechanical performance of PSRCB in each stage of loading. Because ABAQUS software ignores the bond-slip effect between reinforcement and concrete after slab concrete cracking, it indirectly increases the overall stiffness of the structure. Generally, the ABAQUS model can accurately reflect the mechanical performance of the specimen in each stage, which is consistent with the experiment results and proves the dependability of the FE modeling method.

It is discovered that there is a general agreement between the failure mode predicted by the FE model and that observed in the test. Some typical comparisons are shown in Fig. 7. The predicted results indicate that the plastic strain distributed mainly around the loading point and the mid-span section of the beam which is consistent with the formation of the plastic hinge and the bond failure observed in the test.

### Element type, meshes, material settings, loading, and boundary conditions

The concrete and H-beam are modeled using eight-node reduced integration 3D solid elements (C3D8R). Reinforcements and prestressed tendons are modeled with truss elements (T3D2). All steel components are embedded within the concrete slab, and the slippage between the reinforcement and concrete is not considered^40^.

**Figure 6:**
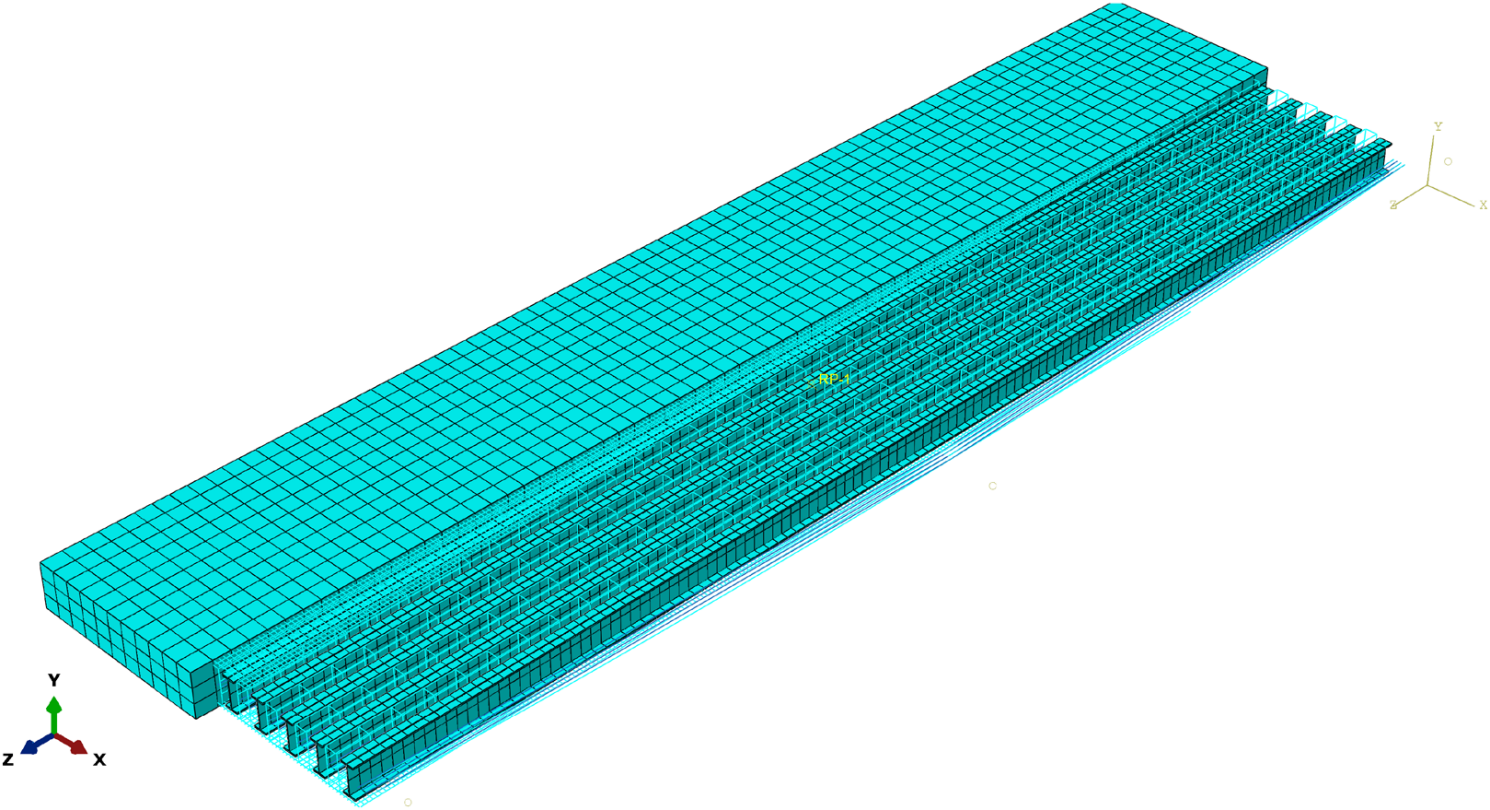
Meshing of the FE model.

Based on the mesh size convergence study (800 mm, 500 mm, 250 mm, and 100 mm), a 500-mm mesh size was employed for the concrete slab to optimize response capture across the entire region and achieve faster computation speed (Fig. 6). For other components, a 300-mm mesh size was utilized for the H-beam, while 500-mm mesh sizes were applied to longitudinal reinforcements and prestressed tendons. An 800-mm mesh size was adopted for stirrup cages, as they are not vertical load-bearing components and are excluded from the analysis content.

The bilinear elastic–plastic stress–strain curve with linear strain hardening is employed to model the H-beam, prestressed tendons, stirrup cages, and reinforcing mesh. When the stress remains within the yield surface, steel exhibits linearly elastic behavior. The Young's modulus (*E*_s_) and Poison's ratio (*ν*_s_) are set to 206,000 N/mm^2^ and 0.3, respectively. If the stress exceeds the yield stress, the hardening modulus *E*′_s_ is taken as *E*′_s_ = 0.01E_s_, in which *E*_s_ is the elastic modulus of the steel^41^. C40 grade concrete is employed for the concrete, Q355 grade steel is used for section steel, and HRB400 grade steel bars are utilized for reinforcement, as specified in^35^. Tensile strength, compressive strength, and yield stress data for concrete and steel can be found in^35^.

In order to simulate the mechanical behavior of PSRCS under overburden pressure, a displacement-controlled analysis was conducted under monotonic loading. As the purpose of this study is to investigate the mechanical mechanism of PSRCS, and to simplify the FE analysis procedure, the boundary condition was assumed to be a simply supported slab, with constraints set on both sides of the PSRCS bottom. Following the constraint method in section "FE model validation", the effects of fixed hinge support and sliding hinge support were achieved. A detailed figure is provided in the Supplementary Data.

## FE model analysis

In this section, the advantages of Prestressed Steel-Reinforced Concrete Structures (PSRCS) over conventional structures, such as Reinforced Concrete Slabs (RCS) and Steel-Reinforced Concrete Slabs (SRCS), are explored by comparing their load–deflection curves, stress distribution within internal components, plastic development behavior of concrete, and other factors. Furthermore, a parametric analysis is conducted to examine the influence of various parameters on the mechanical performance of PSRCS.

### Comparative assessment of results

#### Investigation of load–deflection curves

**Figure 7:**
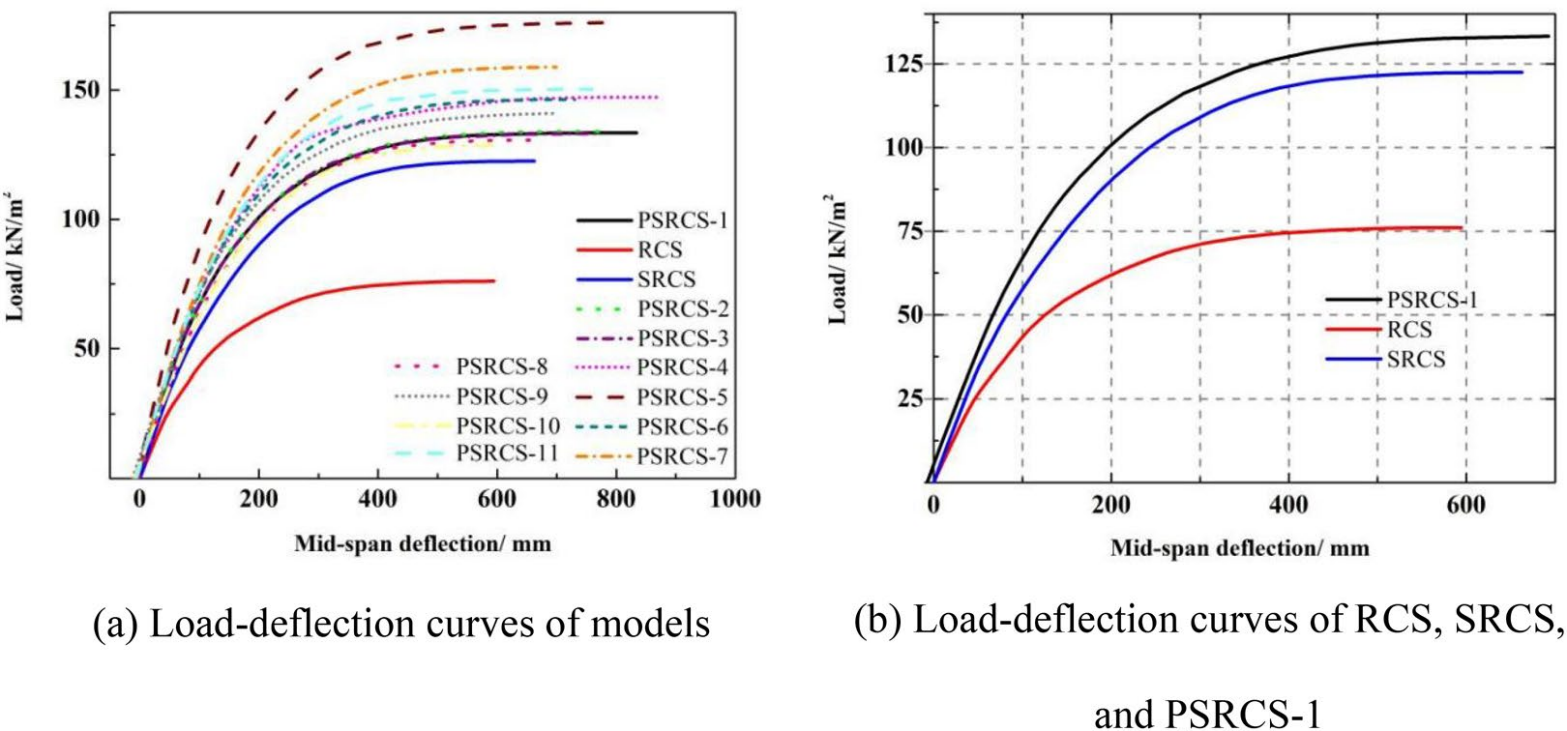
Load–deflection curves of models.

As illustrated in Fig. 7a, the load–deflection curves exhibit similar trends under monotonic loading. Table 2 presents the data results for cracking load (*P*_cr_), cracking deflection (*Δ*_cr_), yield load (*P*_y_), yield deflection (*Δ*_y_), ultimate load (*P*_u_), and ultimate deflection (*Δ*_u_). Compared to RCS and SRCS, PSRCS demonstrates a higher bearing capacity and distinct characteristic points on the load–deflection curve, as shown in Fig. 7b.

**Table 2:** Results of FE models at main stages.

Model	*P*_cr_ (kN/m^2^)	*Δ*_cr_ (mm)	*P*_y_ (kN/m^2^)	*Δ*_y_ (mm)	*P*_u_ (kN/m^2^)	*Δ*_u_ (mm)
RCS	25.0	46.3	60.8	191.0	76.1	591.1
SRCS	35.5	52.9	100.9	237.6	122.5	657.3
PSRCS-1	42.6	54.7	113.4	261.2	133.5	827.1
PSRCS-2	42.4	54.5	109.9	266.5	133.1	876.1
PSRCS-3	42.7	54.8	118.2	259.8	138.8	771.9
PSRCS-4	49.9	58.3	129.7	269.0	147.2	861.2
PSRCS-5	57.1	62.2	155.0	286.5	176.1	899.7
PSRCS-6	44.4	56.1	119.8	240.4	146.3	730.2
PSRCS-7	48.3	58.5	127.9	236.5	158.8	698.9
PSRCS-8	41.7	53.2	110.2	246.1	130.7	671.3
PSRCS-9	48.1	58.3	122.8	268.1	141.3	734.9
PSRCS-10	42.3	54.1	103.6	258.1	128.7	797.9
PSRCS-11	42.9	55.2	130.7	274.7	150.3	833.8

**Figure 8:**
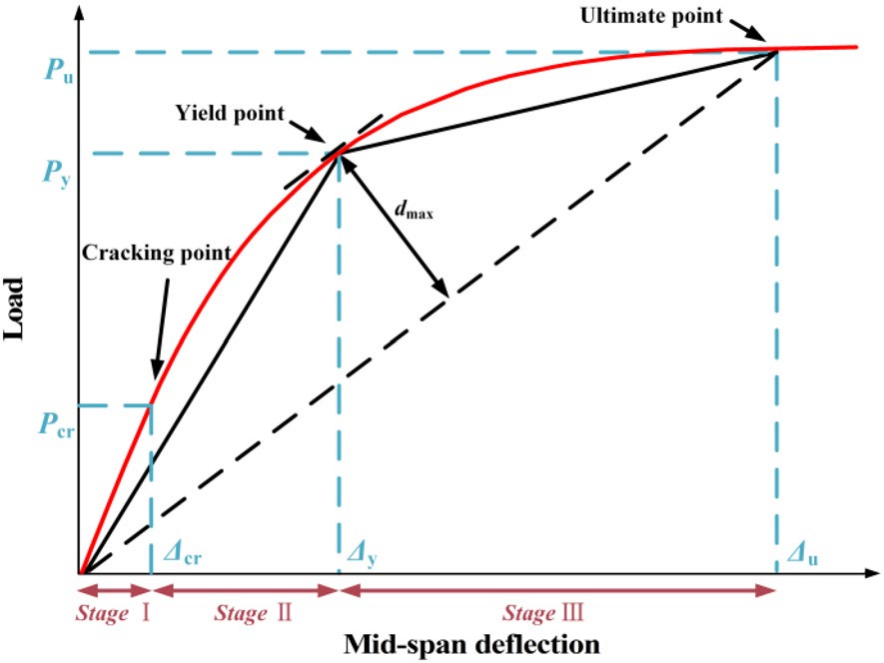
Farthest Point Method for determination of yield points of members.

The cracking point of the model is defined as the inflection point on the load–deflection curve. In this study, the elastic–plastic transition of PSRCS is determined based on the second derivative extremum of its secant stiffness. According to^42^, the yield point of the model is defined as the point of tangency between the load–deflection curve and a line parallel to the line connecting the origin and the ultimate point. The distance from the yield point to the parallel line is defined as '*d*'. When multiple tangent points are present, the point with the largest 'd' value is generally considered as the yield point, as is shown in Fig. 8.

12 $$\left( {\Delta_{{\text{y}}} ,P_{{\text{y}}} } \right) = \mathop {\max }\limits_{{\left( {\Delta_{{\text{y}}} ,P_{{\text{y}}} } \right) = \left( {\Delta ,P} \right)}} d = \frac{{\left| {\Delta \cdot P_{{\text{u}}} - P \cdot \Delta_{{\text{u}}} } \right|}}{{\sqrt {P_{{\text{u}}}^{2} + \Delta_{{\text{u}}}^{2} } }}$$

where (*Δ*, *P*) are the coordinates of any point on the load–deflection curve, (*Δ*_y_, P_y_) are the coordinates of the yield point determined by the farthest point method, (*Δ*_u_, P_u_) are the coordinates of the ultimate point, and 0 ≦ *Δ *≦ *Δ*_u_.

The load of the three models exhibits a linear relationship with the mid-span deflection during the elastic stage. As the three models transition to the elastic–plastic stage, the mid-span deflection of the slabs increases significantly due to the gradual yielding and disengagement of the internal bending members. Upon reaching the yield point, the models enter the plastic stage, and the mid-span deflection of the three models rapidly increases with the load, ultimately reaching the ultimate load. Meanwhile, the ultimate load (*P*_u_) of PSRCS increases by 8.9% and 75.4% when compared to SRCS and RCS, respectively, and the ultimate deflection (*Δ*_u_) of PSRCS increases by 25.8% and 39.9% when compared to SRCS and RCS, respectively.

Compared to RCS, both SRCS and PSRCS exhibit significant improvements in *P*_y_ and *P*_u_, with a more pronounced increase in *P*_cr_ for PSRCS than SRCS. This demonstrates that the incorporation of steel sections and prestressed tendons can substantially enhance the bearing capacity and cracking resistance of the slab. Both SRCS and PSRCS possess the same initial stiffness, which is greater than that of RCS, indicating that the inclusion of H-beams can considerably increase the slab's early flexural stiffness, while the application of prestressed tendons has a minimal effect. The yield ratios of SRCS and PSRCS are higher than RCS, suggesting that the ductility of the slab can be improved through the use of H-beams and prestressed tendons.

#### Investigation of failure modes of concrete and stress of inner components

**Figure 9:**
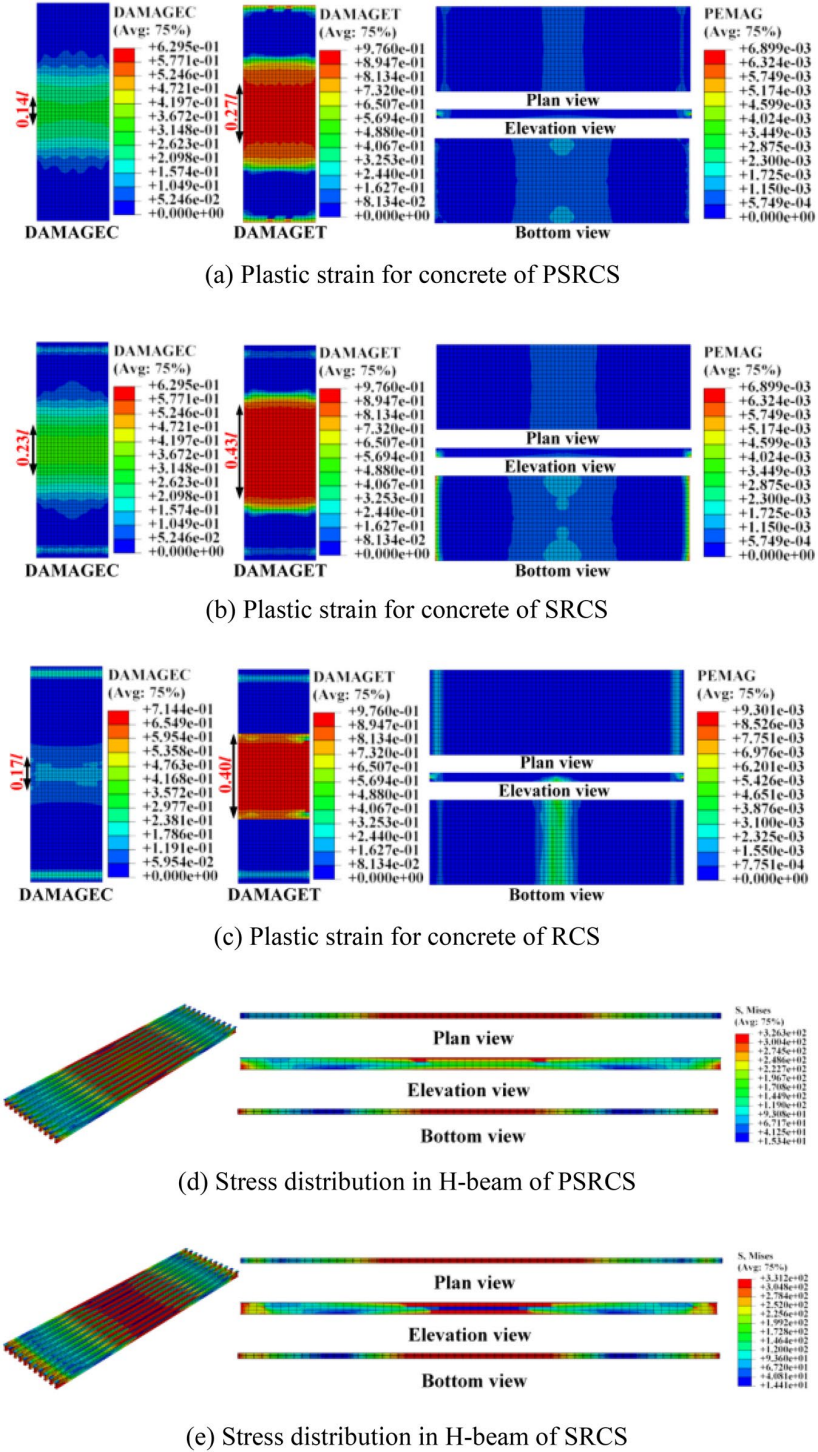

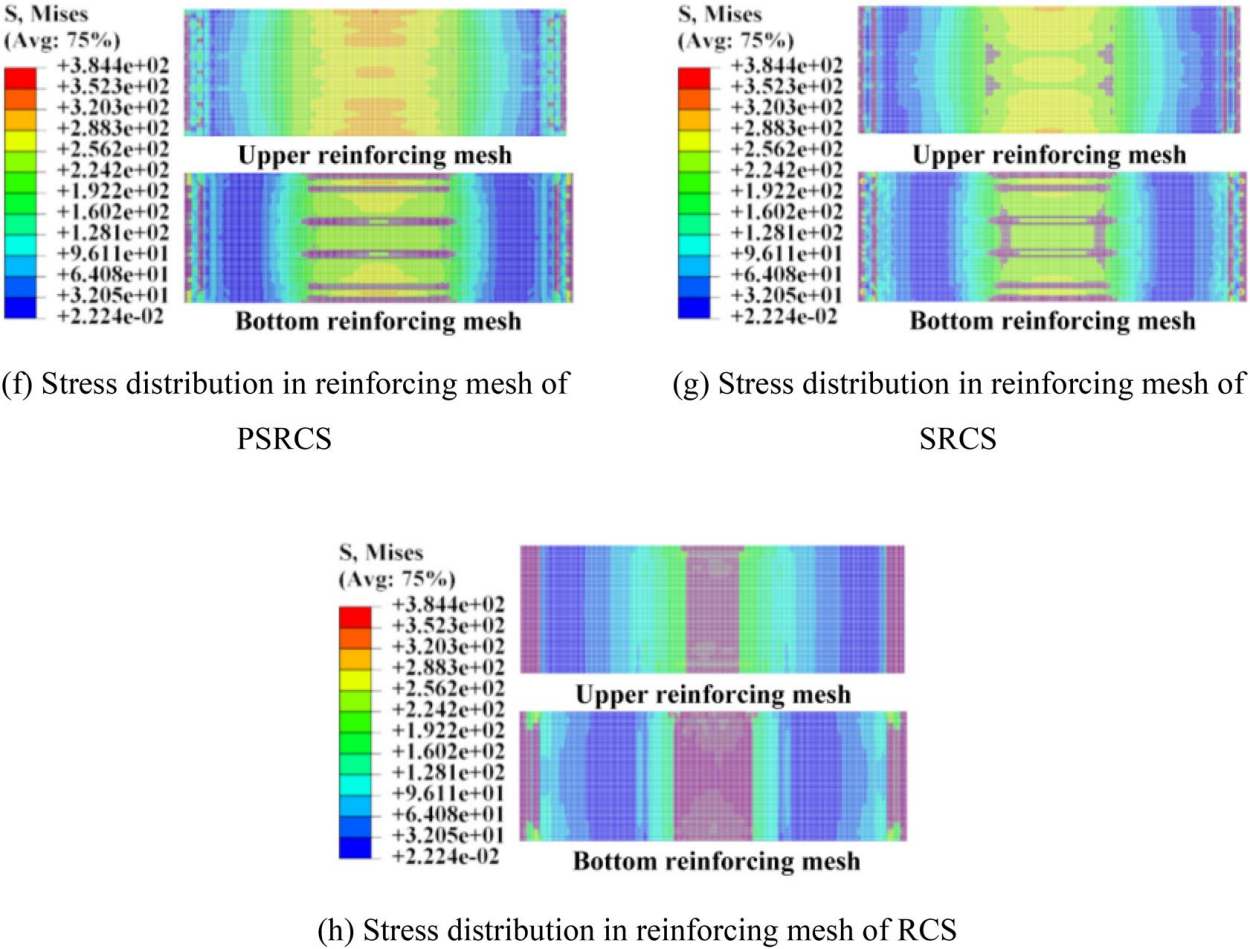
Plastic strain for concrete and stress distribution for inner components.

Upon reaching the ultimate point, three typical plastic strain distribution patterns for concrete models are depicted in Fig. 9a–c. High plastic strain regions are primarily concentrated around the mid-span of the slab, suggesting that the failure of PSRCS is primarily focused at the bottom of the span, consistent with the damage characteristics of flexural structures. Compared to PSRCS, the value of plastic strain (PEMAG) for SRCS and the plastic damage area are larger when reaching the ultimate point. The value of plastic strain for RCS is approximately four times that of PSRCS and is not discussed here. Considering the size of the damage area, *l* represents the span length of the slab, the length of the core region of tensile damage and compressive damage are reduced by 37.2% and 39.1% compared to SRCS, and 32.5% and 17.7% compared to RCS, respectively. This demonstrates that the PSRCS configuration significantly reduces the plastic damage of concrete throughout the entire stage.

As illustrated in Fig. 9d–e, it is evident that the web of the H-beam in PSRCS has not yielded in comparison to SRCS, with the maximum stress of the H-beam reaching 326 MPa, not exceeding the material's yield stress. In contrast, the H-beam in SRCS has yielded, and the maximum stress surpasses the material's yield strength. As demonstrated in Fig. 9f–h, the stress distribution patterns of the reinforcing mesh in PSRCS, SRCS, and RCS are consistent with those of the H-beam, which is not discussed further in this paper.

### Parametric assessment of results

#### Influence of eccentricity of H-beam

**Figure 10:**
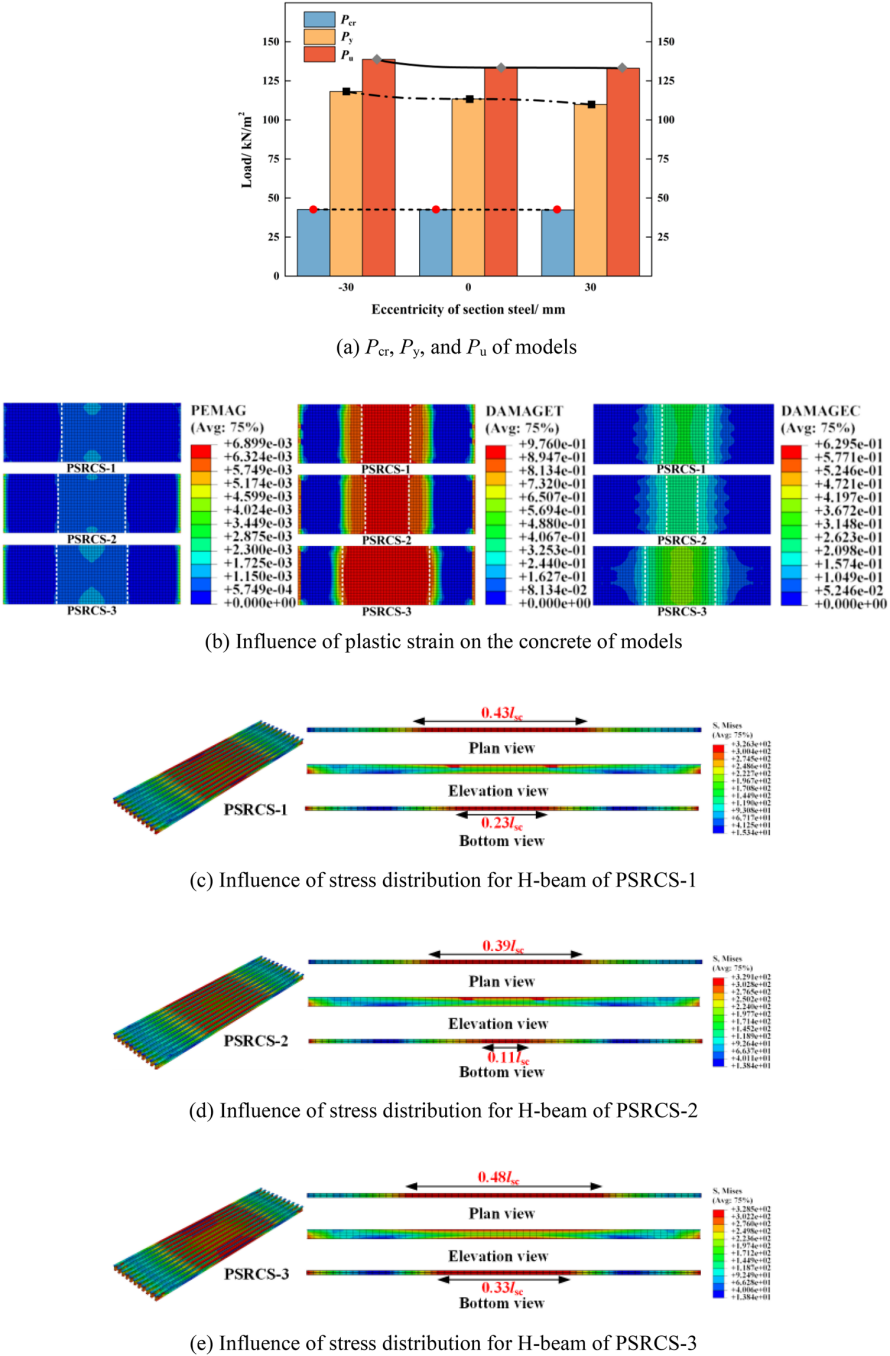
Influence of eccentricity of H-beam.

In numerous engineering applications, the H-beam within bending components does not align with the centroid of the component. As illustrated in Table 2 and Fig. 10a, compared to PSRCS-1, the *P*_cr_, *P*_y_, and *P*_u_ of PSRCS-2 increase by 0.2%, 4.2%, and 3.9%, respectively, while the mid-span deflection of PSRCS-2 decreases by 6.7% upon reaching *P*_u_. In contrast, relative to PSRCS-1, the *P*_cr_, *P*_y_, and *P*_u_ of PSRCS-3 decrease by 0.5%, 3.1%, and 0.3%, respectively, and the mid-span deflection increases by 5.9% when reaching *P*_u_. The bearing capacity increases when the H-beam shifts below the neutral axis, and while the upward shift of the H-beam reduces the bearing capacity, it enhances ductility to a certain extent.

Upon reaching the ultimate load, three typical plastic strain distribution patterns for concrete models are depicted in Fig. 10b. For PSRCS-2, the bottom flange of the section is nearer to the edge of the tensile zone in PSRCS, resulting in a pronounced restraining effect on the tensile zone concrete. Similarly, for PSRCS-3, the high plastic strain distribution region is relatively larger due to the diminished restraint from the bottom flange of the H-beam on the concrete within the tensile zone.

As illustrated in Fig. 10c–e, for PSRCS-2, the high-stress distribution range decreases by 9.3% in the upper flange and 0.52% in the bottom flange compared to PSRCS-1. Likewise, the high-stress distribution range of the H-beam in PSRCS-3 increases by 11.6% and 43.5%, respectively. It can be concluded that the bearing capacity of PSRCS is inversely proportional to the distance between the bottom flange and the edge of the tensile zone. Additionally, the eccentricity of the H-beam has minimal impact on the prestressed tendons, with the variation being negligible. The stress distribution of the reinforcing mesh is similar to that of the H-beam and will not be discussed further in this section.

#### Influence of the span-to-depth ratio

**Figure 11:**
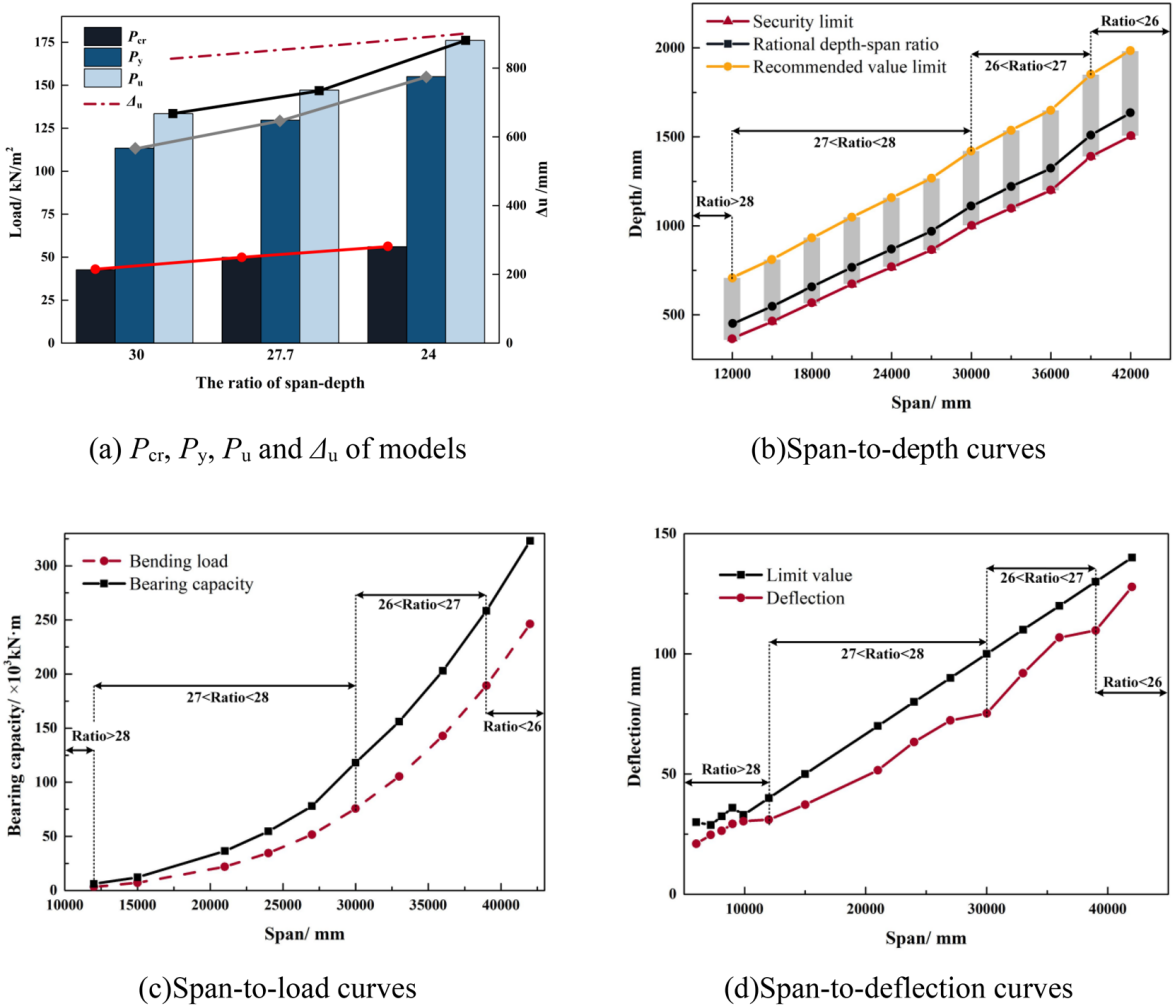
Influence and parametric analysis of the span-to-depth ratio.

The span-to-depth ratio significantly influences the mechanical properties of a structure under various span conditions, necessitating an investigation into an optimal ratio. Figure 11a presents the *P*_cr_, *P*_y_, and *P*_u_ of three models with different span-to-depth ratios. In the elastic stage, the *P*_cr_ of PSRCS-4 and PSRCS-5 increases by 17.1% and 31.6% respectively, compared to PSRCS-1. In the elastic–plastic stage, the *P*_y_ of PSRCS-4 and PSRCS-5 rises by 14.4% and 36.7% respectively, compared to PSRCS-1. In the ultimate state, the *P*_u_ of PSRCS-4 and PSRCS-5 is enhanced by 10.3% and 31.9% respectively, indicating that both *P*_y_ and *P*_u_ of PSRCS significantly increase as the span-to-depth ratio decreases. In the elastic–plastic and plastic stages, the *Δ*_y_ of PSRCS-4 and PSRCS-5 grows by 3.0% and 9.7% respectively, compared to PSRCS-1, and the *Δ*_u_ expands by 4.1% and 8.8%. These results imply that increasing the span-to-depth ratio has a relatively small impact on the ductility of PSRCS but a more substantial effect on its bearing capacity.

To further investigate the impact of the span-to-depth ratio of PSRCS on the span and the upper load, several PSRCS models with different span-to-depth ratios are established to analyze the respective applicable scenarios and determine the most economical and reasonable range. To simplify the analysis, the following provisions are given for the parametric analysis model established in this section:

1. The aspect ratio remains the same as that of PSRCS-1, both being 3:1;
2. The upper load remains constant;
3. As the span lengthens, the span-to-depth ratio of PSRCS changes, and the area of internal reinforcement increases proportionally, but the internal H-beam in the slab retains the same stiffness.

1. The aspect ratio remains the same as that of PSRCS-1, both being 3:1;
2. The upper load remains unchanged;
3. As the span lengthens, the span-to-depth ratio of PSRCS changes, and the area of internal reinforcement increases proportionally, but the internal H-beam in the slab maintains the same stiffness.

Figure 11b presents the span-to-depth curves, indicating that different spans correspond to varying span-to-depth ratios, with the ratio decreasing as the span increases. Three curves depicted in the figure represent the safety curves of the structure, including the minimum limit of the slab thickness, the maximum thickness of the slab suggested based on economic considerations, and the most reasonable recommended curve for the height-thickness ratio, taking both economy and safety index into account. Furthermore, in accordance with the deflection limit specified in "Concrete Structure Design Code" (GB50010-2010)^36^, the range of span-to-depth ratios for PSRCS under different spans is divided, and a suggested ratio is provided, allowing the initial determination of the most applicable slab thickness within a specific span based on this figure.

Figure 11c, d display the span-to-load curves and span-to-deflection curves of PSRCS, determined and analyzed according to the most reasonable ratio suggested in Fig. 8b, respectively. The analysis reveals that when the span is less than 12 m, the bearing capacity of PSRCS closely approximates the mid-span bending moment generated by the upper load, and its deflection also satisfies the deflection limits specified in "Concrete Structure Design Code" (GB50010-2010)^36^. While the bearing capacity of PSRCS increases significantly faster than the upper load as the span lengthens, the bearing capacity of PSRCS experiences an increase when the span-to-depth ratio changes, reaching a plateau at the critical value of the ratio in the span-to-deflection curves.

#### Influence of the ratio of longitudinal reinforcement and prestressed tendons

**Figure 12:**
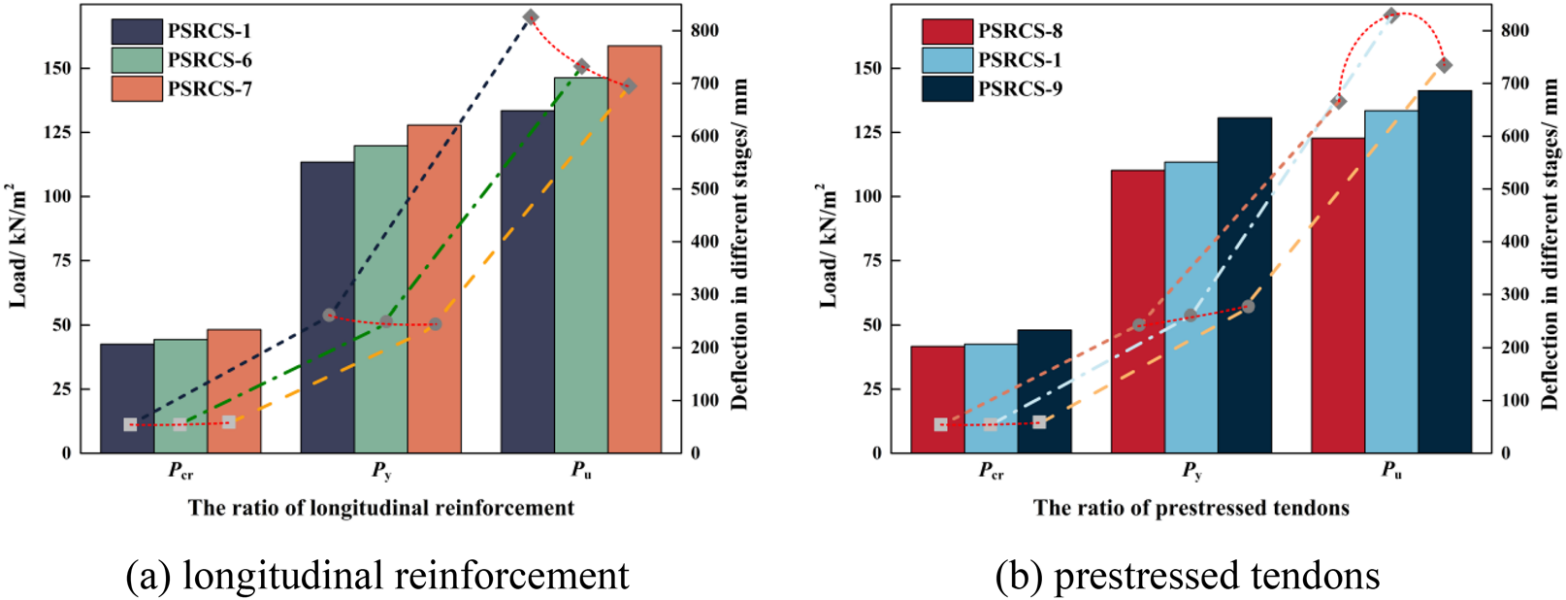
*P*_cr_, *P*_y_, and *P*_u_ of models.

Figure 12 presents the *P*_cr_, *P*_y,_ and *P*_u_ of three models with varying ratios of longitudinal reinforcement and prestressed tendons. In the elastic stage, the *P*_cr_ of PSRCS-6, PSRCS-7, and PSRCS-9 increase by 4.2%, 13.3%, and 12.9%, respectively, compared to PSRCS-1, while PSRCS-8 decreases by 2.1%. In the elastic–plastic stage, the *P*_y_ of PSRCS-6, PSRCS-7, and PSRCS-9 increase by 5.6%, 12.8%, and 8.3%, while PSRCS-8 decreases by 2.8% compared to PSRCS-1. In the ultimate state, the *P*_u_ of PSRCS-6, PSRCS-7, PSRCS-8, and PSRCS-9 are enhanced by 9.6%, 19.0%, 2.1%, and 5.8% compared to PSRCS-1, respectively. It can be concluded that the *P*_cr_, *P*_y,_ and *P*_u_ are significantly improved by increasing the ratio of longitudinal reinforcement and prestressed tendons. Although increasing the longitudinal reinforcement ratio has a more visible effect on bearing capacity, the ratio of prestressed tendons should not be neglected in the design.

To determine the optimal reinforcement ratio, *Δ*_cr_, *Δ*_y,_ and *Δ*_u_ are analyzed for the three models. In the elastic stage, *Δ*_cr_ increases with the ratio of longitudinal reinforcement and prestressed tendons. The increase in *Δ*_cr_ when raising the ratio of reinforcement is smaller compared to *P*_cr_, which implies that longitudinal reinforcement and prestressed tendons do not maintain the same yield rate after reaching the elastic–plastic stage. The increase in longitudinal reinforcement on the variation of structural failure features, making PSRCS more susceptible to brittle failure.

The *Δ*_u_ of the three models did not follow the same growth trend as *P*_u_. With the increase of the reinforcement ratio, the *Δ*_u_ of longitudinal reinforcement demonstrates a significant decreasing trend, indicating that increasing the ratio of longitudinal reinforcement significantly reduces the deflection of PSRCS in the ultimate stage. Consequently, the concrete in its compressive zone reaches the ultimate compressive strain first and is rapidly damaged, which aligns with the characteristics of over-reinforcement damage. The *Δ*_u_ variation curve of prestressed tendons is fitted and analyzed, showing that when the ratio of prestressed tendons is approximately 0.3%, the bearing capacity and ductility of PSRCS are in an optimal state.

**Figure 13:**
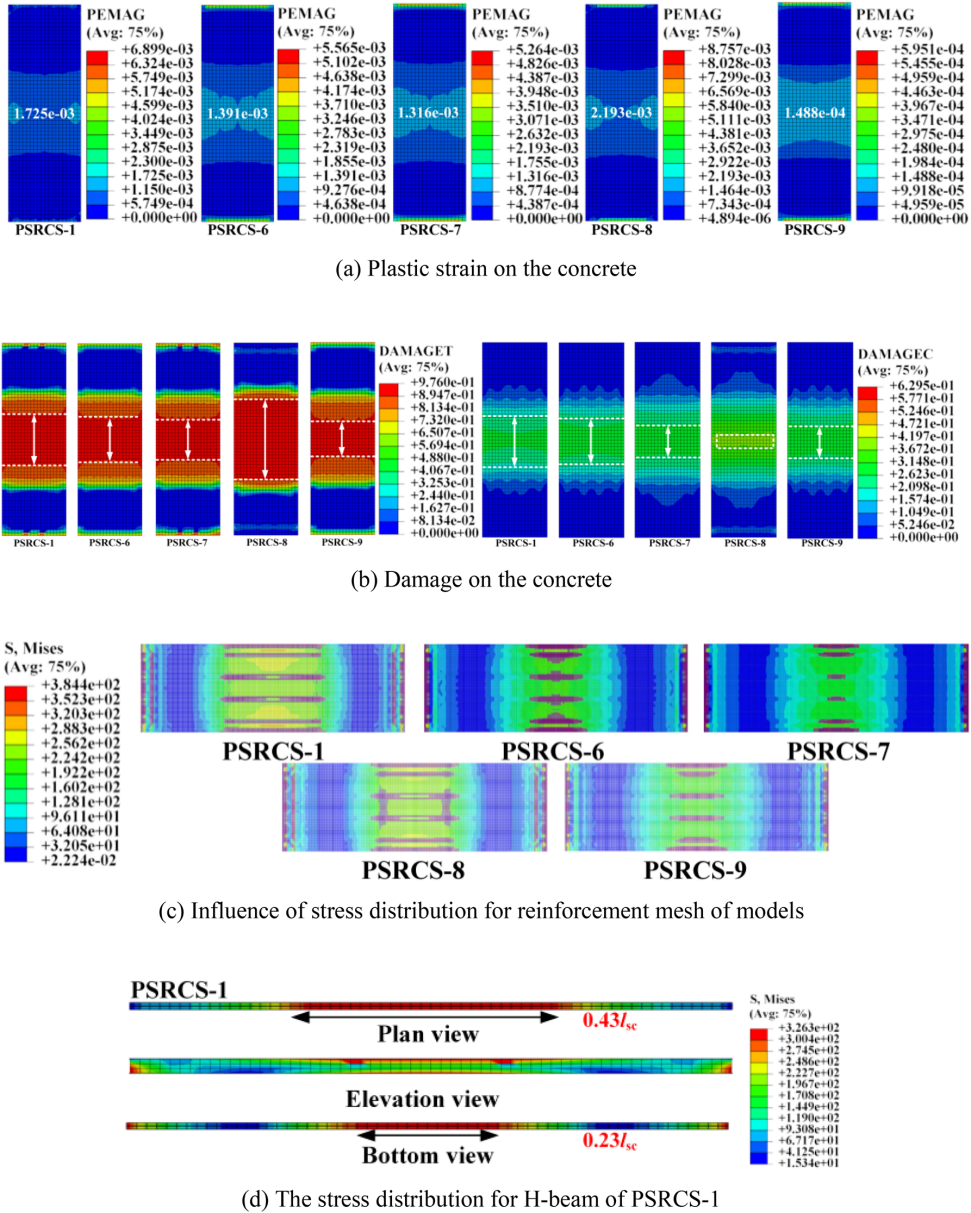

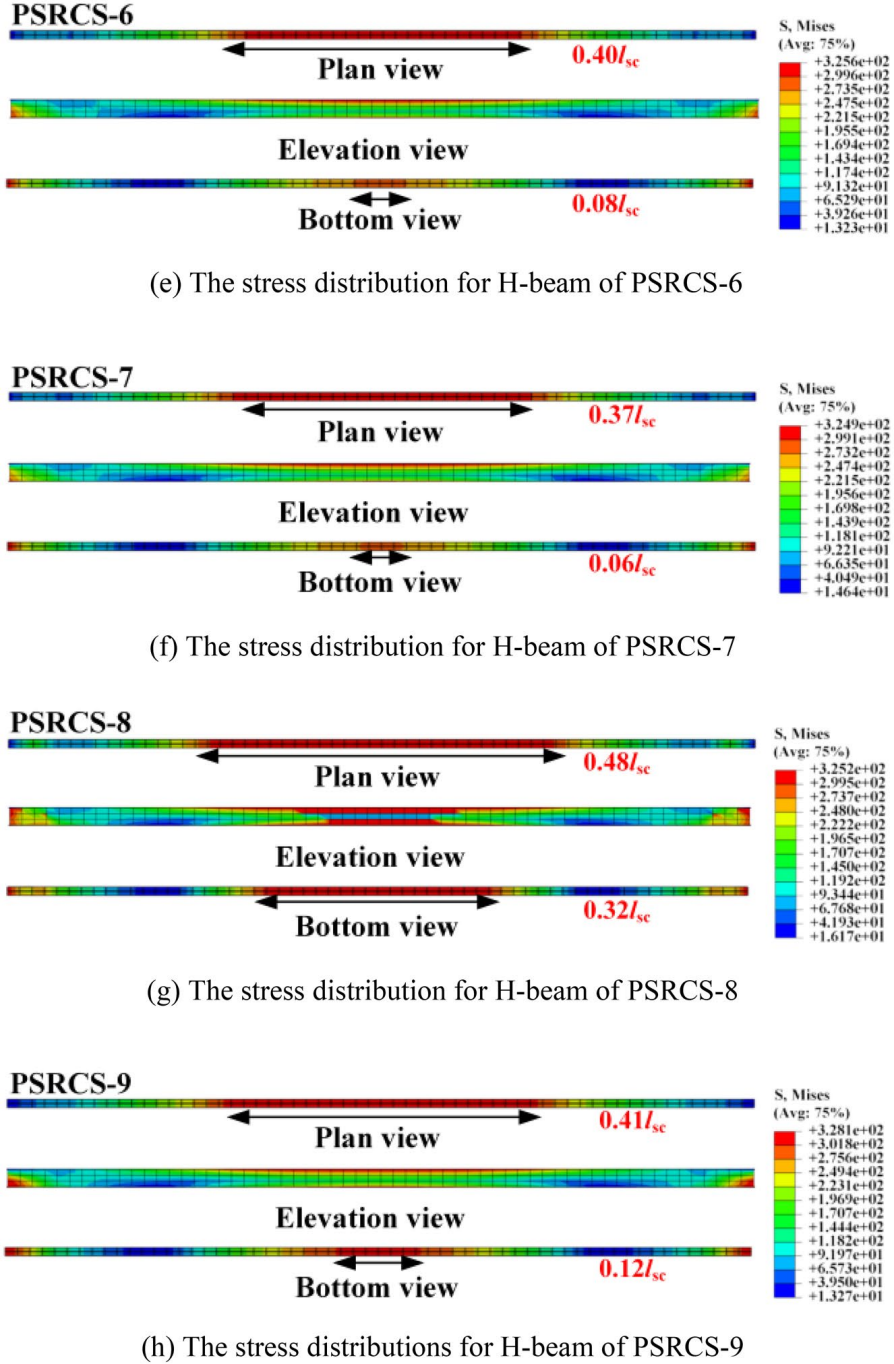
Concrete damage and stress distributions for inner components of models.

The impact of plastic strain on concrete at different reinforcement ratios when PSRCS reaches *P*_u_ is illustrated in Fig. 13a, b. As the ratio of longitudinal reinforcement and prestressed tendons increases, the plastic strain values at the bottom of the slab decrease correspondingly. It can be concluded that increasing the ratio of longitudinal reinforcement has a limited effect on the tensile and compressive damage of the slab. Furthermore, increasing the ratio of prestressed tendons may effectively reduce damage in the core region and delay the cracking of PSRCS, highlighting the significance of prestressed tendons in controlling crack development and concrete damage.

As illustrated in Fig. 13c–h, increasing the ratio of longitudinal reinforcement effectively reduces stress distribution in the mid-span region of the reinforcing mesh and significantly decreases stress magnitude and distribution in the tensile region of the bottom flange of the H-beam, while its influence in the compressive region of the upper flange is relatively minor. Increasing the ratio of prestressed tendons can moderately reduce stress distribution in the mid-span region of the reinforcing mesh but has relatively subtle effects on the stress distribution and magnitude of the H-beam. It can be concluded that variations in the ratio of prestressed tendons have a similar effect to those of longitudinal reinforcement, which can enhance the bearing capacity of PSRCS to a certain degree.

#### Influence of the ratio of steel content

**Figure 14:**
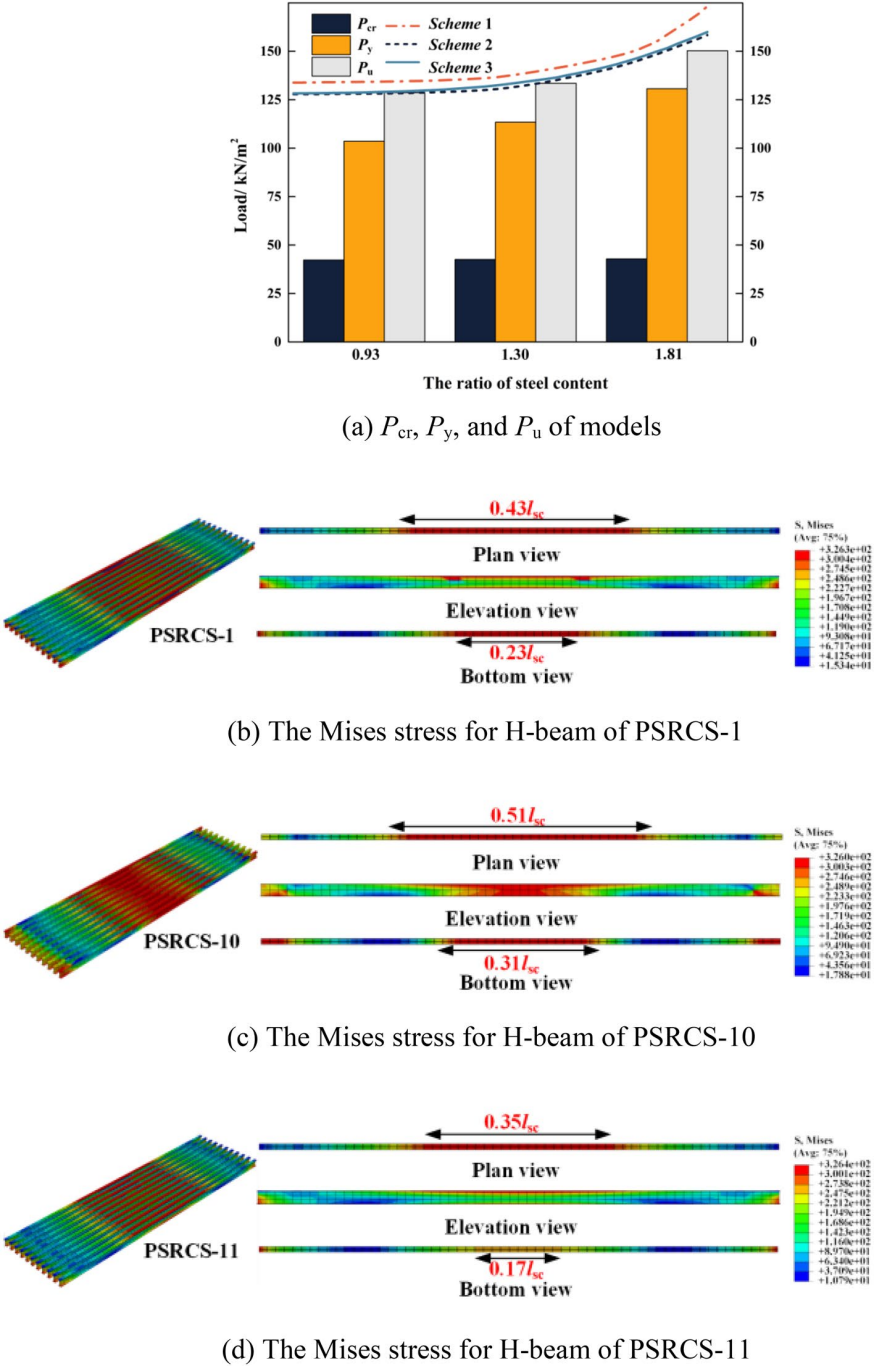
Influence of the steel content ratio.

The *P*_cr_, *P*_y,_ and *P*_u_ of the three models with different ratios of steel content are shown in Fig. 14a. Compared with PSRCS-1, the *P*_cr_ of PSRCS-10 has a 0.7% decrease in the elastic stage, while the *P*_cr_ of PSRCS-11 has a 0.7% improvement, the *P*_y_ of PSRCS-10 has an 8.6% decrease in the elastic–plastic stage, while the *P*_y_ of PSRCS-11 has a 15.3% improvement. In the ultimate stage, the *P*_u_ of PSRCS-10 has a 3.6% decrease compared to PSRCS-1, while PSRCS-11 has a 12.6% increase. It can be concluded that by increasing the steel content of the H-beam in proportion, the *P*_y_ and *P*_u_ of PSRCS are significantly increased, and the increase of *P*_y_ and *P*_u_ is greater with the increase of steel content, which indicates that the steel content is more effective in improving the bearing capacity of PSRCS in elastic–plastic and plastic stages.

Figure 14a presents the *P*_cr_, *P*_y,_ and *P*_u_ values for the three models with varying steel content ratios. Compared to PSRCS-1, PSRCS-10 exhibits a 0.7% decrease in *P*_cr_ during the elastic stage, while PSRCS-11 demonstrates a 0.7% improvement. In the elastic–plastic stage, the *P*_y_ of PSRCS-10 decreases by 8.6%, whereas PSRCS-11 increases by 15.3%. In the ultimate stage, PSRCS-10's *P*_u_ decreases by 3.6% compared to PSRCS-1, while PSRCS-11 sees a 12.6% increase. These results suggest that increasing the steel content of the H-beam proportionally leads to significant enhancements in *P*_y,_ and *P*_u_ for PSRCS. Furthermore, the improvement in *P*_y,_ and *P*_u_ becomes more pronounced as steel content increases, indicating that steel content is highly effective in augmenting PSRCS bearing capacity during elastic–plastic and plastic stages.

Figure 14b–d illustrates the stress distribution of the H-beam. The yield section length of the upper flange in PSRCS-10 increases by 18.6% compared to PSRCS-1, while the bottom flange experiences a 34.8% increase. In contrast, the upper flange yield section in PSRCS-11 is 18.6% shorter than that in PSRCS-1, and the bottom flange does not yield at the mid-span. These findings suggest that raising the steel content ratio effectively diminishes the high-stress region distribution within the section during the yielding stage. Moreover, it decelerates the degradation of H-beam stiffness and substantially enhances the structure's overall bearing capacity. This analysis does not consider tensile longitudinal reinforcements and other remaining elements.

To further investigate the variation of steel content, Scheme 0 is defined for PSRCS-10 and PSRCS-11 to adjust the steel content ratio by modifying the H-beam's full section area. In this section, three additional schemes are established to examine various factors influencing the steel content variance. Scheme 1 alters the upper and bottom flange areas of the section, Scheme 2 adjusts the web area, and Scheme 3 modifies the number of H-beams by regulating the total section area, as illustrated in the Supplementary Data.

As illustrated in Fig. 14a, FE analysis is conducted to examine the steel ratio variation in the other three schemes, and the corresponding steel content ratio-*P*_u_ curves for each scheme are fitted. Scheme 1 exceeds Scheme 0, Scheme 2, and Scheme 3 in each stage, indicating that augmenting the flange thickness can more effectively enhance the bearing capacity of PSRCS. Scheme 2 is smaller than Scheme 0 at each level, demonstrating that the increase in web thickness does not substantially contribute to the bearing capacity despite the additional steel usage. Scheme 3 is nearly identical to Scheme 0, suggesting that the overall section area in the tensile zone determines the bearing capacity. This can be attributed to the fact that ABAQUS neglects bond-slip between steel and concrete. In practical applications, the load-bearing capacity of Scheme 3 during the elastic–plastic stage is increased due to the larger contact area. This will be further demonstrated in subsequent experiments.

Utilizing the theoretical calculation method outlined in section "Theoretical calculations of bearing capacity, section stiffness, and deflection of PSRCS", the comparison results between the calculated bearing capacity and deflection of PSRCS and the finite element (FE) simulation values are presented in Table 3.

**Table 3:** Comparison between theoretical calculation and FE simulation.

Model	*M*_1_ (kN·m)	*M*_2_ (kN·m)	*M*_2_/*M*_1_	*y*_1_ (mm)	*y*_2_ (mm)	*y*_2_/*y*_1_
PSRCS-1	2719.74	2933.35	1.079	244.34	261.2	1.069
PSRCS-2	2838.86	3010.66	1.061	245.85	266.5	1.084
PSRCS-3	2638.79	2855.35	1.082	237.91	259.8	1.092
PSRCS-4	3109.82	3354.58	1.079	229.72	269.0	1.171
PSRCS-5	3416.44	3584.57	1.049	248.70	286.5	1.152
PSRCS-6	2873.24	3091.90	1.076	226.37	240.4	1.062
PSRCS-7	3067.50	3289.89	1.072	217.37	236.5	1.088
PSRCS-8	2642.27	2850.33	1.079	231.30	246.1	1.064
PSRCS-9	2944.08	3106.21	1.055	244.84	268.1	1.095
PSRCS-10	2481.02	2681.53	1.081	230.24	258.1	1.121
PSRCS-11	3133.81	3358.45	1.072	242.67	274.7	1.132

Where *M*_1_ means the theoretical calculation results of bending moment, *M*_2_ means the FE results of bending moment, *y*_1_ means the theoretical calculation results of deflection, *y*_2_ means the FE results of deflection.

Table 3 reveals that the average value of *M*_y_/*M*_tc_ for the 13 models is 1.071, while the average value of *Δ*_y_/*Δ*_tc_ for the 13 models is 1.103. The theoretical calculation and FE simulation results exhibit a close and well-matched relationship, indirectly verifying the accuracy of the PSRCS model establishment and the feasibility of the calculation theory.

## Conclusions

This study introduced an innovative prestressed steel reinforced concrete slab (PSRCS) designed for heavy loads and large spans. The advantages of this structure were evaluated through theoretical research and finite element model analysis. Based on the investigations, the following key findings and contributions can be highlighted:

1. The study proposed relevant theoretical formulas applicable to the innovative PSRCS, including formulas for bearing capacity under various situations, section stiffness, and deflection both before and after deformation.
2. The efficacy of the established model was validated using ABAQUS, demonstrating that PSRCS has superior bearing capacity and fracture resistance compared to traditional reinforced concrete slabs (RCS) and steel reinforced concrete slabs (SRCS).
3. The comprehensive parametric analysis of the PSRCS using ABAQUS, assessing the effects of H-beam eccentricity, prestressed degree, and reinforcement ratio on bearing capacity and internal stress components.
4. Based on parameter analysis, the span-to-depth ratio range of PSRCS under different spans was divided, with security limit (minimum slab thickness) and economic value limit(maximum slab thickness) provided. Rational recommended ratios for different spans were also given, offering a design basis for PSRCS application across various spans.

These findings provide valuable insights and a solid foundation for further development and application of PSRCS in various engineering projects. Future research could concentrate on carrying out vertical loading tests on PSRCS (currently underway) and comparing the results with the theoretical calculation formulas and finite element analysis presented in this study. Additionally, investigating further design parameters and optimization methods to enhance the performance and cost-efficiency of PSRCS would be valuable.

## Supplementary Information

Supplementary Information.

## Data Availability

The data that support the findings of this study are available from the first author, [Tiancheng Han], upon reasonable request.
