# Supplementary material for: An investigation of the flexural behaviour of large-span prestressed and steel-reinforced concrete slabs
Source: Sci Rep. 2023 Jul 3;13:10710. doi: 10.1038/s41598-023-37137-6 (PMC10318042; doi:10.1038/s41598-023-37137-6)
Supplement: Supplementary file 1 — Supplementary Information. [file 41598_2023_37137_MOESM1_ESM.docx]

**Vertical mechanic performance analysis of large-span prestressed steel reinforced concrete slab**

Tiancheng Han^a,b^, Shuting Liang^a,b^, Xiaojun Zhu^c^, Wenkang Wang^a,b^, Jian Yang^a,b^

^a^School of Civil Engineering, Southeast University, Nanjing, China, 210096;

^b^Key Laboratory of Concrete and Pre-stressed Concrete Structures of the Ministry of Education, Nanjing, China, 210096;

^c^Architecture Design and Research Institute Ltd, Southeast University, Nanjing, China, 210096

Corresponding Author: Shuting Liang (e-mail: stliang@seu.edu.cn )

**3.3 Element Type and Meshes**

The concrete is modeled using eight-node reduced integral format 3D solid elements (C3D8R). The reinforcements are modeled using truss elements (T3D2). For section steel, a four-node conventional shell element with reduced integration elements (S4R) is employed. Both the longitudinal reinforcements and the stirrup cages are embedded with concrete slab. Slippage between the reinforcement and the concrete is not taken into account(Yang, Liang, Zhu, & Dang, 2021). The meshing of the FE model is shown in Supplementary data.

| 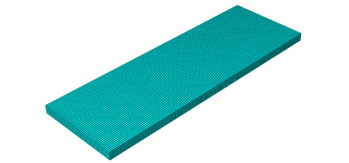 | 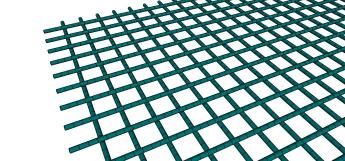 | 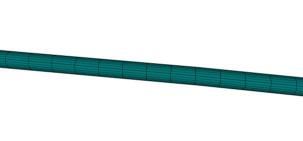 | 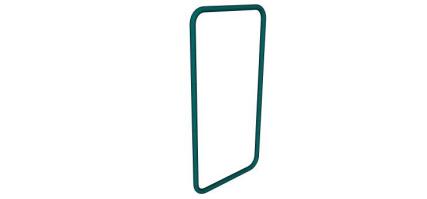 |
| --- | --- | --- | --- |
| (a) Concrete | (b) Reinforcing mesh | (c) Prestressed tendons | (d) Stirrup cages |
| 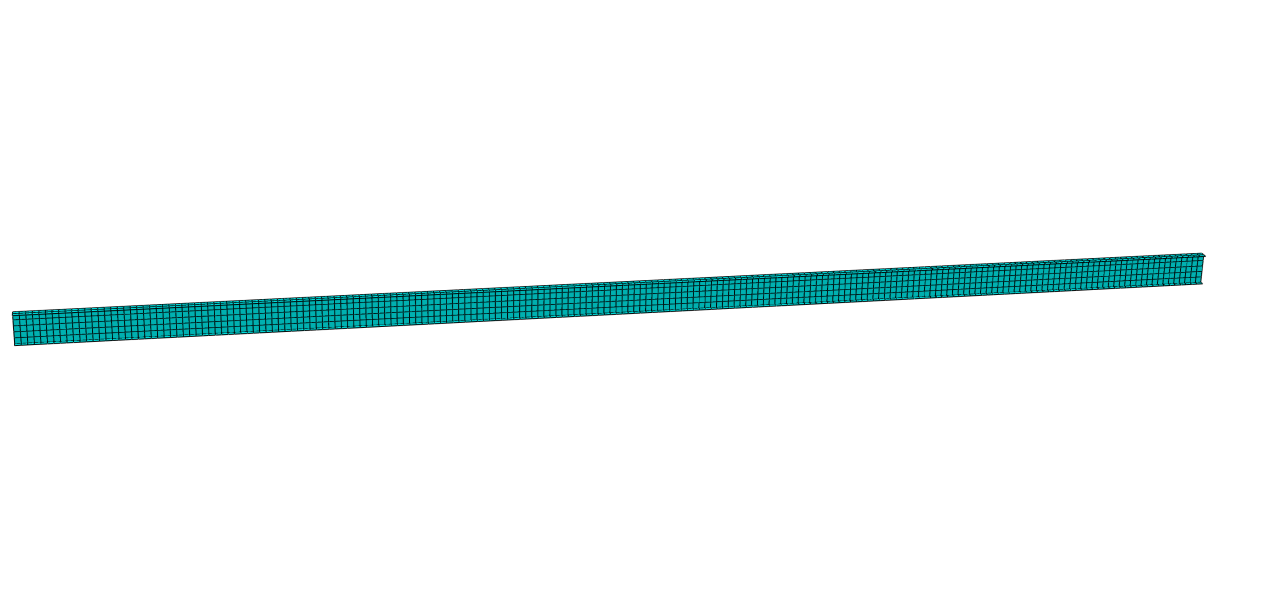 | | | |
| (e) Section steel | | | |

Supplementary fig. 1 Meshing of the FE model

**3.5 Loading and Boundary Conditions**

To simulate the mechanical behaviors of PSRCS under the overburden pressure, a pressure-controlled analysis has been conducted under monotonic loading. Ensuring that PSRCS can achieve the yield plateau, the FE loading procedure adopts a stepwise loading method, starting from 0 and gradually increasing by 20 kN/m^2^ in each step. To simplify the FE analysis procedure, the boundary condition is assumed to be a simply supported slab by setting constraints on both sides of the bottom of the PSRCS, so the effects of fixed hinge support and sliding hinge support are achieved. The detailed figure is shown in Supplementary Data.


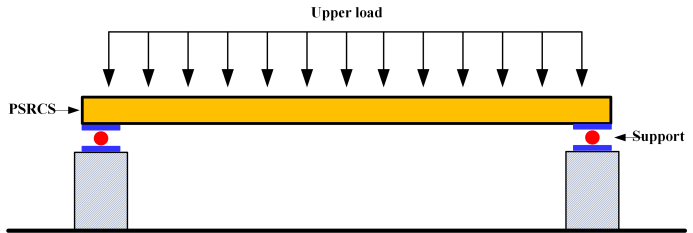


Supplementary fig. 2 Loading and constraints of the PSRCS

**4.2.4 Influence of the ratio of steel content**

To make further efforts in the variation of steel content, scheme 0 is defined for PSRCS-10 and PSRCS-11 to adjust the ratio of steel content by altering the area of the full section of section steel. In this section, three more schemes are established to investigate the various factors influencing the variance of steel content. Scheme 1 changes the upper and bottom flange area of the section, scheme 2 changes the web area, and scheme 3 changes the number of section steel by regulating the total section area, as shown in Fig. 4.


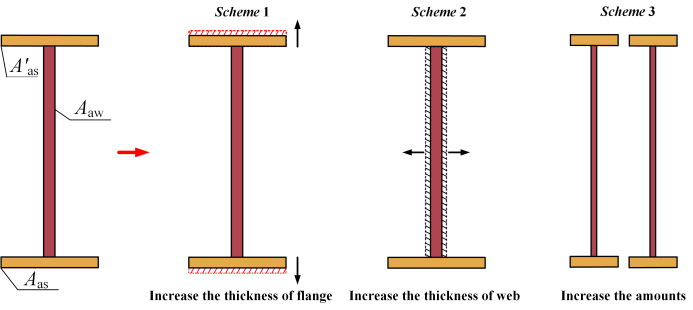


Supplementary fig. 3 Schemes for the ratio of steel content change
